# Sulfur‐Rich Sustainable Copolymers for Enhancing Redox Kinetics and Alleviating Cathode Passivation in Lithium‐Sulfur Batteries

**DOI:** 10.1002/EXP.20240447

**Published:** 2026-05-26

**Authors:** Sangeeta Sahu, Arnab Ghosh, Monisha Monisha, Murali Krishna, Shakir Ali Siddiqui, Sunan Tian, De‐Yi Wang, Sagar Mitra, Bimlesh Lochab

**Affiliations:** ^1^ Department of Chemistry School of Natural Sciences Shiv Nadar Institution of Eminence Gautam Buddha Nagar India; ^2^ IMDEA Materials Institute Getafe Spain; ^3^ Department of Energy Science and Engineering Indian Institute of Technology Bombay Mumbai India

**Keywords:** cardanol, ion‐diffusion coefficients, lithium‐sulfur batteries, organic redox mediators, organosulfur cathodes, sulfur copolymers

## Abstract

Lithium‐sulfur (Li‐S) batteries are promising candidates for advanced energy storage systems. However, their performance is hindered by uncontrolled cathode passivation due to the formation of electronically insulating lithium sulfide (Li_2_S). Here, we report a sulfur‐rich copolymer, poly(sulfur‐*random*‐cardanol cystamine) [poly(S‐*r*‐Ccys)], as an efficient cathode material that enables spatially regulated Li_2_S growth and improved redox kinetics. The nitrogen and oxygen functionalities in the Ccys moiety facilitate electrostatic interactions with lithium polysulfides, enhancing their dissolution and redistribution. Pulsed‐field gradient nuclear magnetic resonance measurements confirm improved Li^+^ ion diffusion, while galvanostatic intermittent titration technique analysis reveals faster reaction kinetics in the poly(S‐*r*‐Ccys) cathode. The poly(S‐*r*‐Ccys) cathodes exhibit superior cycling stability compared to conventional elemental sulfur cathodes, maintaining 76.7% of their initial capacity after 300 cycles at 1 C, with a low average capacity fade of just 0.077% per cycle. This work demonstrates that poly(S‐*r*‐Ccys) offers a viable strategy to overcome Li_2_S deposition challenges and improve the cycle life of Li‐S batteries without altering the conventional ether‐based electrolyte system.

## Graphical abstract

A molecularly engineered, sustainable sulfur‐rich redox copolymer cathode delivers accelerated sulfur conversion and suppressed passivation, enabling high sulfur utilization and durable, high‐performance LiS batteries.

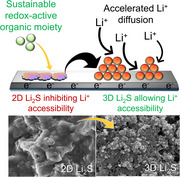

## 1 Introduction

The persistent environmental catastrophes and recent health and geopolitical crises exacerbate concerns about renewable energy storage and utilization prerequisites. Consequently, the exigency for rechargeable high‐energy‐density batteries is becoming more crucial [1]. Lithium‐sulfur (Li‐S) batteries with exceptionally high theoretical energy density (∼2600 Wh kg^−1^) are the potential alternative to contemporary lithium‐ion batteries [2, 3, 4].

The conventional Li‐S batteries typically include a Li metal anode, a sulfur/carbon composite cathode, and an ether‐based electrolyte [3, 4, 5]. During the discharge of a Li‐S battery, elemental sulfur (S_8_) is reduced to lithium sulfide (Li_2_S), transitioning through intermediate lithium polysulfides (Li_2_S*_x_*, 2 ≤ *x* ≤ 8), whereas the reverse process occurs during the charging process [3]. The lithium polysulfides (LiPS), including Li_2_S_8_, Li_2_S_6_, and Li_2_S_4_, readily dissolve into the ether electrolytes, and consequently, their electrochemical interconversion becomes associated with liquid‐phase reactions with rapid kinetics. Benefiting from such fast redox kinetics, Li‐S batteries exhibit high reversible specific capacity during the initial few cycles [6]. However, the sparingly solvating characteristic of Li_2_S in the ether electrolyte with intermediate donor number leads to a pervasive growth of highly insulating Li_2_S [7, 8] over the surface of sulfur cathodes causing passivation, which is believed to be one of the main reasons for the premature failure of the battery [9, 10, 11].

In general, the electrolyte‐soluble LiPS serve as the intrinsic redox mediators to regulate the deposition/dissolution behavior of Li_2_S [12]. However, during the charge–discharge cycling of Li‐S batteries, the soluble LiPS are continuously consumed. Therefore, such intrinsic redox mediacy of LiPS is restricted, especially in lean LiPS conditions, either at the end of the discharge process or at the beginning of the charging [13]. Numerous heterogeneous redox mediators including metals [14, 15], metal oxides [11, 16, 17, 18], metal nitrides [19, 20], hybrid composites of metal oxides/nitrides [21, 22], metal sulfides [23], MXenes [24], have been studied to regulate the desired spatial distribution of discharge products over the cathode, and thereby, facilitate the oxidation of Li_2_S. Despite the improved battery performance realized by these heterogeneous mediators, the associated mediation process simultaneously relies on the nature of the electronically conductive surface of carbonaceous materials, as fillers, in the sulfur cathodes. However, these materials tend to get covered by a sheath of insulating Li_2_S layer after the initial few cycles and gradually lose their activities of controlling three‐dimensional growth of Li_2_S in sulfur cathodes [25, 26]. However, organic mediators based on heteroatom‐bearing small molecules [25, 26, 27] and polymers [28, 29, 30, 31, 32] have shown promising results in overcoming these challenges. Tuning the solvation structures of polysulfide anions by selecting high‐donor‐number electrolyte solvents [33, 34] and salts [35, 36] is found to be a promising alternative strategy to control the spatial deposition and favorable growth of the end‐discharge product independent of such heterogeneous mediators. Nevertheless, such electrolyte compositions with high Gutmann donor numbers inevitably undergo parasitic reactions with metallic Li anode, and therefore, their utilization in Li‐S batteries is practically limited [37, 38]. Recently, redox mediators have also been studied to enhance the specific capacity of Li‐S batteries by assisting the homogenous growth of higher‐dimensional Li_2_S [27, 39, 40, 41, 42, 43]. However, such redox mediators may experience gradual deactivation and often result in severe parasitic reactions with Li anode through their shuttling [44, 45, 46]. Regulating the growth of Li_2_S by dissolving it into electrolytes through weak electrostatic interactions with an additive is seemingly a feasible approach that inevitably avoids the requirement of a sophisticated, complex cathode structure. For instance, ammonium cation, produced by the dissolution of ammonium salts in high‐donicity solvents such as dimethyl sulfoxide (DMSO) and tetramethylene sulfone (SL), promotes the dissolution of Li_2_S by strongly interacting with S^2−^ anion via hydrogen bonding (N─H*^δ^* ^+^─S^2^) [47, 48]. However, the vulnerability of the Li anode against high‐donicity solvent molecules (DMSO and SL) persists. Unfortunately, the sparingly solvating nature of ammonium salts in the low‐donicity 1,2‐dimethoxyethane and 1,3‐dioxolane solvents imparts a challenge for the complete dissolution of Li_2_S [49]. Interestingly, He et al. [50]. demonstrated the solubilization effect of trifluoroacetamide (TFA) on Li_2_S in the typical DME/DOL‐based electrolyte. Nevertheless, the presence of highly acidic hydrogens raises the question of the chemical compatibility of TFA and induces corrosiveness in the anode. Therefore, an imperative shift is underway for promoting the dissolution of Li_2_S without compromising the composition of the standard ether electrolyte (i.e. 1 m solution of lithium bis(trifluoromethanesulfonyl)imide salt in the 1:1 (v/v) mixture of DME and DOL for Li‐S batteries. Alternatively, a benign‐by‐design approach for such mediators is desired to enable sustainability in the fabricated battery with improved performance.

In this endeavor, we explore a versatile benzoxazine (Bz) monomer that provides the opportunity to enable the incorporation of multiple hetero‐atoms in a one‐step, atom‐economic manner via a typical Mannich‐like condensation reaction. Cardanol (C, cashew nutshell agro‐waste) and bio‐origin cystamine (cys) with characteristic disulfide bond (─S─S─) are employed first to synthesize cardanol‐cystamine (Ccys) monomer. Bulk copolymerization of the as‐synthesized Ccys monomer with elemental sulfur (petroleum industry by‐product, annual production >60 million tonnes) results in poly(sulfur‐*random*‐cardanol cystamine), hereinafter referred to as poly(S‐*r*‐Ccys), which has been explored as a cathode material in Li‐S battery. The intrinsic functional groups comprising hetero atoms (i.e. N and O) present in the poly(S‐*r*‐Ccys) copolymer are envisioned to promote the dissolution of Li_2_S by offering electrostatic interactions and thereby improving the spatial control of sulfur species deposition on the cathode. The higher solubility of Li_2_S in the presence of the Ccys moiety leads to the formation of discrete Li_2_S particles through a controlled precipitation/nucleation mechanism similar to that observed in lithium‐oxygen batteries [51]. The pulsed‐field gradient (PFG) NMR technique is widely recognized as an essential tool for characterizing lithium dendrite formation in rechargeable lithium metal batteries; however, its application in Li‐S batteries has been relatively unexplored. In this study, we employ the PFG‐NMR technique to establish a correlation between Li^+^ ion‐diffusion coefficients (*D*_Li_^+^) in the solutions of Li_2_S with poly(S‐*r*‐Ccys) and their mutual interactions while varying the relative amount of the sulfur copolymer. Ex‐situ scanning electron microscopy (SEM) characterization of the fully discharged poly(S‐*r*‐Ccys) cathode confirms the formation of discrete Li_2_S particles with a three‐dimensional porous morphology that inevitably provides a continuous steady percolation pathway for Li^+^ ions into the bulk of poly(S‐*r*‐Ccys) cathode during battery operation, leading to a stable performance of Li‐S battery with excellent Coulombic efficiency [52]. The galvanostatic intermittence titration technique (GITT) experiments reveal superior electrochemical kinetics of the poly(S‐*r*‐Ccys) cathode, and the theoretical calculations further corroborate this observation.

## 2 Results and Discussion

### 2.1 Synthesis and Characterization of the Monomer and Copolymer

**SCHEME 1:**
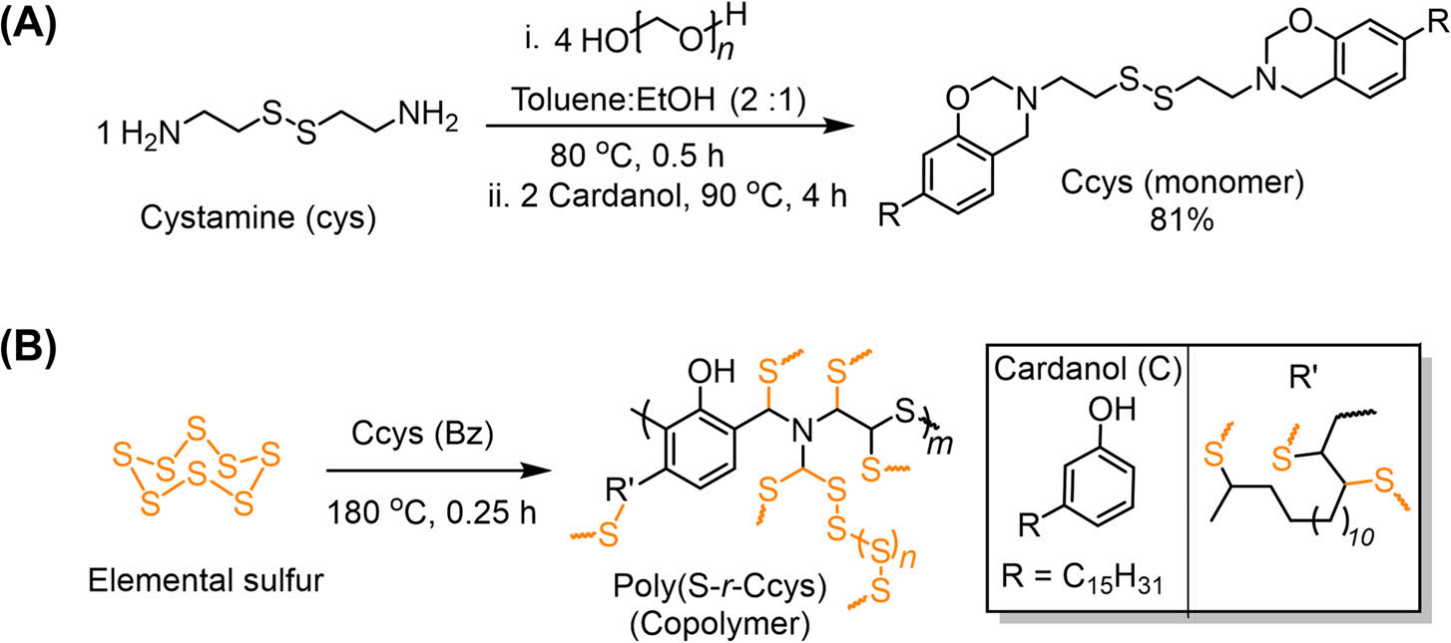
(A) Synthesis of Ccys benzoxazine monomer and (B) copolymerization reaction of Ccys with elemental sulfur to form poly(S‐*r*‐Ccys) copolymer.

The cardanol cystamine (Ccys) Bz monomer was synthesized using biobased feedstocks cardanol (saturated) and cystamine (Scheme 1A). The formation of a reactive oxazine ring and the methylene groups in the long alkyl R‐chain in the Ccys monomer offers multiple reactive sites for the polysulfane diradicals to form the poly(S‐*r*‐Ccys) copolymer (Scheme 1B).

**FIGURE 1:**
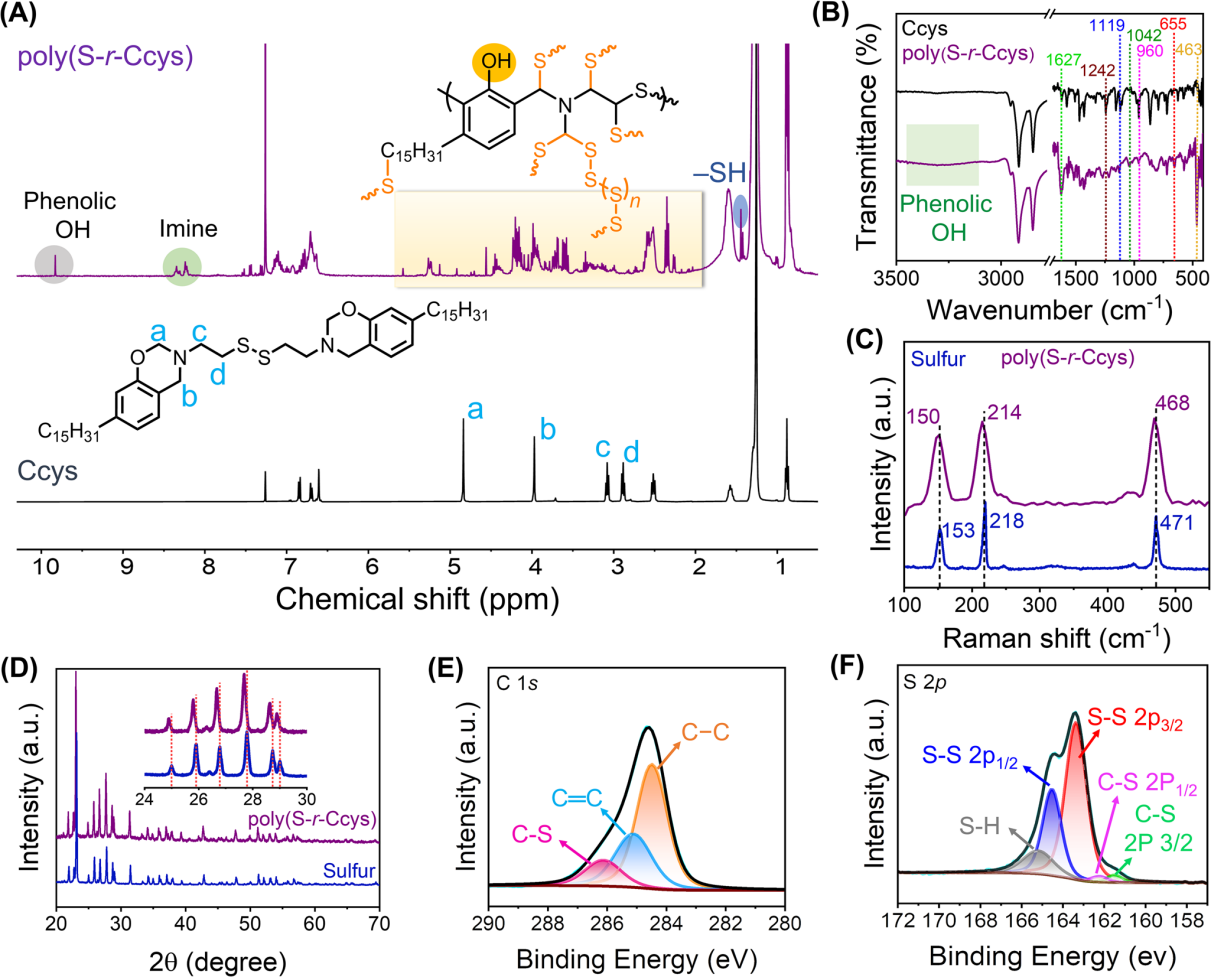
(A, B) Comparison between NMR and FTIR spectra of Ccys monomer and poly(S‐*r*‐Ccys) copolymer, (C, D) comparison of Raman spectra and powder XRD patterns of sulfur and poly(S‐*r*‐Ccys), (E, F) deconvoluted C1s and S 2p XPS spectra of poly(S‐*r*‐Ccys) copolymer.

The successful formation of the Ccys monomer and the poly(S‐*r*‐Ccys) copolymer and their chemical structures were confirmed by various characterization techniques. The characterizations of the monomer (^13^C and HRMS, high‐resolution mass spectra) and the copolymer (^13^C and DEPT) are represented in detail (Figures S1 and S2). The ^1^H (Figure 1A) and ^13^C NMR (Figure S1B) spectra revealed significant changes in both the aliphatic and aromatic regions of Ccys after the inverse vulcanization process. Copolymerization with elemental sulfur resulted in minimal perturbation of the methyl proton (carbon) signal at *δ* = 0.9 ppm (^1^H) and 14 ppm (^13^C), corresponding to the long aliphatic chain of Ccys, indicating the preservation of this structural motif. In contrast, the characteristic oxazine ring ^1^H (^13^C) signals assigned to protons **a** and **b** at *δ* = 3.97 ppm (51.14 ppm) and 4.85 ppm (82.56 ppm), respectively, disappeared in the copolymer spectrum, confirming ring‐opening during the reaction. A new resonance at *δ* = 1.44 ppm, attributed to thiol protons [53], emerged alongside multiple new signals in the *δ* = 2.3–5.5 ppm (^1^H) and 22.0–81.0 ppm (^13^C) regions, consistent with the formation of CH_2_─S_x_ and N─CH_2_─S_x_ linkages in the poly(S‐*r*‐Ccys) network. Additionally, the formation of N─(CH_2_)_2_─S─S bonds was supported by signals at *δ* = 3.08 and 2.88 ppm (^1^H) and 50.33 and 38.08 ppm (^13^C), respectively. Significant changes in the aromatic region of the ^1^H NMR spectrum further suggest reactions of polysulfane diradicals with the aromatic ring. DEPT NMR analysis revealed the formation of Mannich and *N,O*‐acetal linkages, evidenced by the appearance of new methylene (negative signals at *δ* = 22.0–81.0 ppm), methine (positive signals at *δ* = 17, 115–134, and 195 ppm), methyl (positive signal at *δ* = 30 ppm), and quaternary carbon signals (*δ* = 144–173 ppm), indicating extensive covalent integration of sulfur chains at multiple reactive sites. Moreover, sulfane radical‐induced oxazine ring‐opening is corroborated by the appearance of a characteristic imine functionality, along with distinct ^1^H and ^13^C NMR signals at approximately 8.2 ppm and 171 ppm, respectively.

The FTIR spectrum of the Ccys monomer exhibits the characteristic bands of C─O─C bond stretching (asymmetric and symmetric) and C─N─C bond stretching (asymmetric and symmetric) at 1237, 1120, 1042, and 960 cm^−1^, respectively, due to the oxazine ring (Figure 1B). The absence of these characteristic bands and the appearance of a new peak at 655 and 463 cm^−1^ due to the C─S and S─S [54] bond and the peaks at 1627 cm^−1^ and ∼3300 cm^−1^ due to imine and phenolic ─OH functional groups further corroborate the opening of oxazine rings by polysulfane diradicals to initiate copolymerization reactions. The phenolic ─OH and N‐containing functional groups [55] in the copolymer are anticipated to coordinate with Li^+^ ions and provide opportunities for Li^+^ ions accessibility at the vicinity of the sulfur chains of poly(S‐*r*‐Ccys) backbone and thereby might improve the rate capability of the sulfur copolymer. Figure S3 presents the gel permeation chromatography (GPC) profile of the sulfur copolymer, exhibiting a broad elution peak centered at 22.7 mL. The polymer displayed a weight‐average molecular weight (*M*_w_) of 2003 Da and a dispersity (*Đ*) of 1.4, indicating a moderately narrow molecular weight distribution. The formation of the low molecular weight sulfur copolymer is attributed to efficient chain‐transfer reactions facilitated by the high concentration of S─S bonds within the polymer matrix. These dynamic covalent interactions limited chain propagation, thereby suppressing the development of high‐molecular‐weight species. This mechanistic interpretation is consistent with the findings reported by Yang et al. [56]. Furthermore, the molecular weight distribution observed in GPC is corroborated by MALDI‐TOF mass spectrometry (Figure S4), which confirmed the predominance of low‐molecular‐weight species. Raman spectra of elemental sulfur and the poly(S‐*r*‐Ccys) are represented in Figure 1C. The shift of characteristic Raman peaks of S─S─S bending and S─S stretching vibrations to lower wavenumber with an anharmonic peak broadening for poly(S‐*r*‐Ccys) can be accounted for the variation in intermolecular interactions at the surface and the quantum confinement [57]. Powder XRD patterns were recorded to corroborate this observation. As evident from the inset of Figure 1D, the diffraction peaks of the copolymer were relatively broad and slightly shifted to a lower 2*θ* value compared to the elemental sulfur. The crystallite size of poly(S‐*r*‐Ccys) was estimated to be 52 nm, relatively smaller than that of elemental sulfur (63 nm). It is speculated that the charge transfer kinetics for composite electrodes comprised of active materials with smaller crystallites become superior due to their larger surface‐area‐to‐volume ratio [58], and consequently exhibit superior rate capabilities, especially at higher current densities [59].

The wide‐survey scan XPS spectrum reveals the elemental composition of poly(S‐*r*‐Ccys) copolymer (Figure S5). The deconvoluted C 1s XPS spectrum consists of three characteristic peaks, which are ascribed to aromatic sp^2^ C═C, aliphatic C─C, and C─S bonds (Figure 1E), indicating inverse vulcanization of elemental sulfur in the presence of Ccys monomer to form poly(S‐*r*‐Ccys) copolymer. The XPS spectrum of elemental sulfur exhibited a characteristic spin–orbit doublet, with S 2p_1/2_ and S 2p_3/2_ peaks located at 164.2 eV and 165.4 eV, respectively, consistent with the presence of unbound sulfur species (Figure S6). In contrast, the copolymer spectrum (Figure 1F) revealed a shift of the S─S doublet to lower binding energies (163.3 and 164.5 eV), indicative of covalently incorporated sulfur within the polymer matrix. Additionally, new peaks observed at 161.4 and 162.1 eV were assigned to C─S bonds, confirming the formation of covalent linkages between sulfur chains and the organic framework. These spectral features collectively demonstrate the presence of chemically distinct sulfur environments, highlighting the unique structural configuration of the copolymer. The additional peak component at the binding energy of 165.2 eV is ascribed to the thiol functionality in the copolymer [60].

**FIGURE 2:**
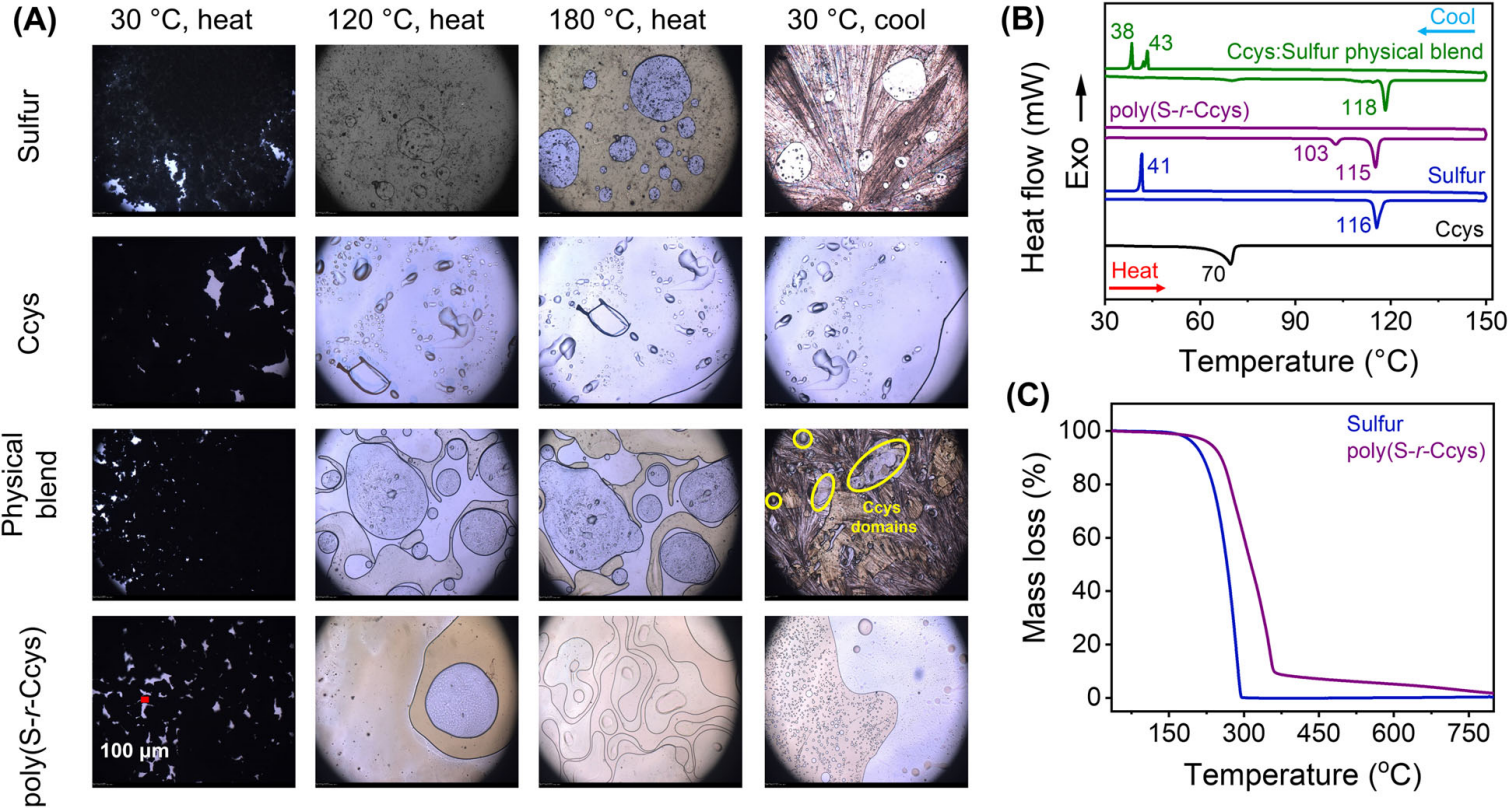
(A) HSM images captured during the heating and cooling cycle, (B) DSC heat‐cool cycle of elemental sulfur, Ccys, poly(S‐*r*‐Ccys), and sulfur/Ccys (9:1) physical blend, (C) TGA thermogram of elemental sulfur and poly(S‐*r*‐Ccys) copolymer.

As a preliminary test to rule out the existence of elemental sulfur impurity in the copolymer, hot stage microscopy images (HSM) of poly(S‐*r*‐Ccys), elemental sulfur, Ccys, and their physical blend (1:9, same as the mass ratio in copolymer) were captured at different temperatures. The temperature‐dependent HSM images of the copolymer demonstrate a remarkable difference in morphology in comparison to elemental sulfur, Ccys monomer, and their physical blend during heating/cooling (Figure 2A). The physical blend of sulfur and Ccys exhibited isolated localized melt domains of Ccys (circled yellow) in between the crystalline domains of sulfur. Interestingly, the poly(S‐*r*‐Ccys) revealed a homogeneous single phase during heating and cooling, confirming the absence of unreacted sulfur in the copolymer and the consumption of feed‐in sulfur in the formation of the copolymer. This evinces the capability of the 10 wt% Ccys monomer to covalently attach with the high amount (90 wt%) of sulfur during the inverse vulcanization process. SEM image of the as‐synthesized poly(S‐*r*‐Ccys) copolymer revealed an average particle size ranging from 10–20 µm (Figure S7). However, to diminish the diffusion length of Li^+^ ions into the active material and thereby increase the active material utilization, the particle size of the copolymer was reduced by ball‐milling during fabrication of the poly(S‐*r*‐Ccys) cathode, as described in the Experimental Section. Differential scanning calorimetry (DSC) measurements were performed to understand thermal transitions due to the copolymerization of elemental sulfur with the Ccys monomer (Figure 2B). The DSC thermogram of Ccys and elemental sulfur show the melting endotherm transitions at 70°C and 116°C, respectively. However, the elemental sulfur exhibits a cold crystalline exotherm at 41°C. The DSC thermogram of poly(S‐*r*‐Ccys) reveals distinctive endotherm transitions at 103°C and 115°C, ratifying the HSM results. No exothermic transition is noticed during the cooling of poly(S‐*r*‐Ccys), manifesting the absence of elemental sulfur impurity in the copolymer and the existence of carbon‐bonded polysulfur chains in the copolymer. A lowering in melting temperature of the copolymer compared to elemental sulfur further supports the existence of smaller crystallites in the former due to the Gibbs–Thomson effect [61]. Thermogravimetric analysis (TGA) confirmed a very high sulfur loading in the copolymer (Figure 2C), which agrees with the feed‐in ratio. A distinct mass loss pattern of the physical blend at the same weight ratio as the prepared copolymer (Figure S8A) confirmed the existence of sulfur as organo‐polysulfides in poly(S‐*r*‐Ccys). To probe it further, derivative thermogravimetric (DTG) traces were analyzed (Figure S8B). The DTG analysis reveals a difference in mass loss behavior of the constituent units (Ccys and sulfur), physical blend, and copolymer. Sulfur shows a single mass loss peak with a maximum at 286°C and a physical blend at 268°C, which shows the predominance of elemental sulfur mass loss patterns. While the copolymer clearly shows multiple mass loss signatures ascribed to the existence of different chain lengths of sulfur covalently linked with the organic units.

### 2.2 Influence of Poly(S‐*r*‐Ccys) on Li_2_S Solubility

**FIGURE 3:**
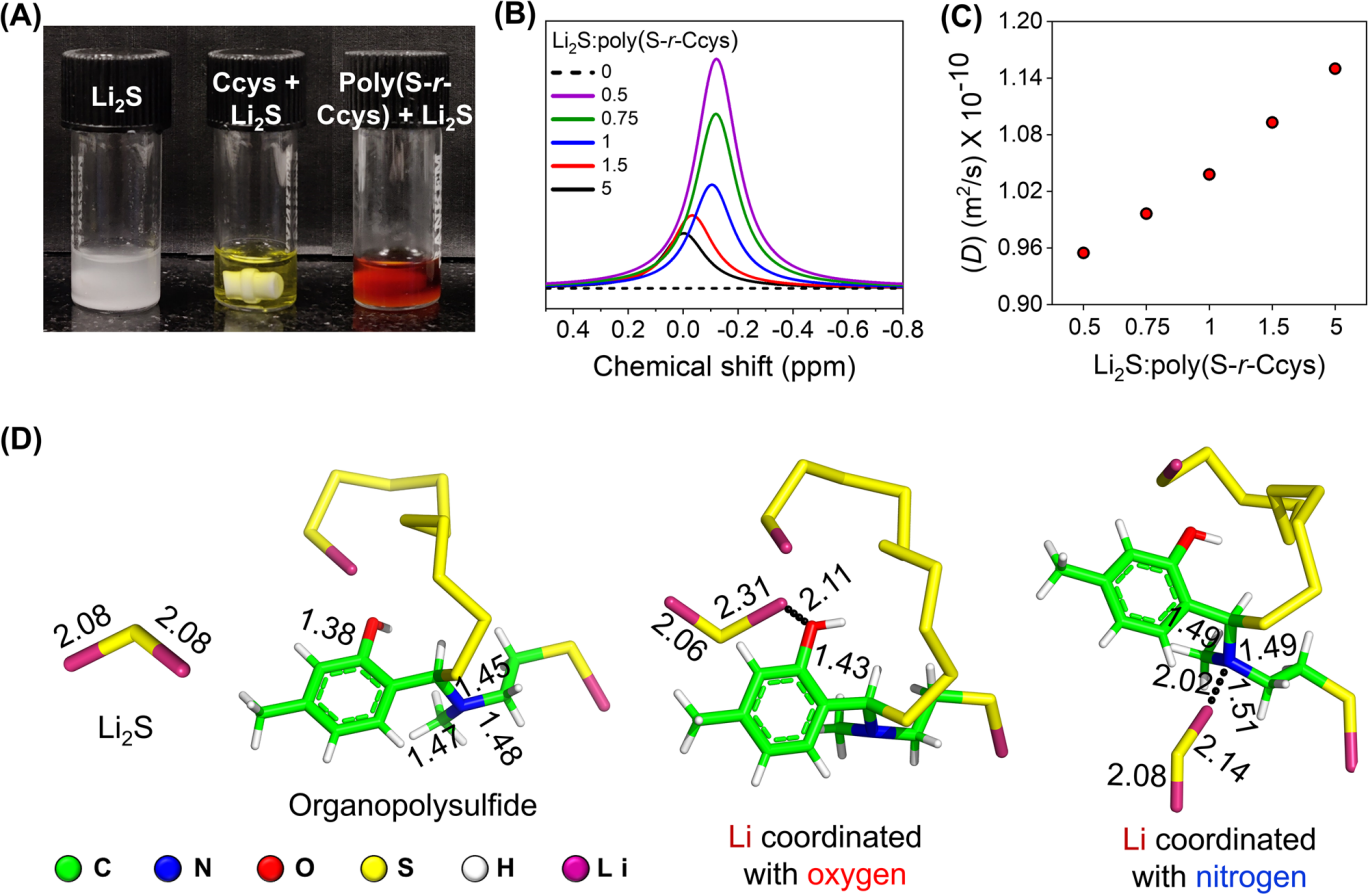
(A) Digital image of Li_2_S solubility in the base electrolyte with and without copolymer and monomer, (B) concentration‐dependent Li_2_S dissolution study with varying amounts of copolymer in Li_2_S, (C) Li^+^ ion diffusion coefficient by varying amounts of poly(S‐*r*‐Ccys) in Li_2_S obtained by PFG NMR, (D) theoretical models for the interaction of Li_2_S with a representative organo‐polysulfide.

Heteroatoms containing organic molecules as electrode additives endow the battery with stability and high efficiency by minimizing electrode passivation and promoting the solubility‐assisted controlled growth of Li_2_S [62]. In this study, the effect of poly(S‐*r*‐Ccys) copolymer on the solubility of Li_2_S in ether electrolyte was examined by NMR studies. Li_2_S exhibits sparingly soluble characteristics in the ether solvents comprising a 1:1 (v/v) mixture of 1,2‐dimethoxyethane and 1,3‐dioxolane (Figure 3A). The white turbid suspension of Li_2_S in ether electrolyte was changed to a transparent yellow and transparent red solution after adding Ccys and poly(S‐*r*‐Ccys) separately. The observed variation in coloration upon the addition of the monomer and copolymer to the Li_2_S solution in DME/DOL is likely due to the formation of distinct lithium polysulfide species. In the case of the copolymer, coordination interactions between Li^+^ ions and functional groups such as phenolic‐O, amine‐N, and polysulfide sulfur atoms may stabilize specific polysulfide intermediates. These interactions can induce bathochromic shifts in the absorption spectrum, resulting in the observed red coloration. This phenomenon is consistent with previous reports demonstrating the role of heteroatom coordination in modulating polysulfide speciation and optical properties [63]. Furthermore, such coordination environments are also expected to impart redox mediator functionality, as suggested by Zhao et al. [27]. potentially enhancing electrochemical performance. These observations indicate that the individual Ccys and poly(S‐*r*‐Ccys) could significantly increase the solubility of Li_2_S in the ether electrolyte, possibly due to chemical interactions between Li_2_S and Ccys or poly(S‐*r*‐Ccys). Concentration‐dependent ^7^Li NMR spectra of Li_2_S solution with varying amounts of poly(S‐*r*‐Ccys) were recorded to gain insight into the interactions of Li_2_S with poly(S‐*r*‐Ccys). A noticeable upfield shift in the broad ^7^Li NMR signal, from *δ* = 0.00 ppm to −0.120 ppm was observed (Figure 3B), which could be ascribed to a considerable interaction between Li^+^ center in Li_2_S and the heteroatoms (i.e. O and N) rich solvation sphere provided by poly(S‐*r*‐Ccys) and as a consequence of shielding effect and a probable change in the Li^+^ ion coordination number [64]. The successful coordination of Li^+^ with the poly(S‐*r*‐Ccys) copolymer was additionally supported by a decrease in the Li^+^ ion‐diffusion coefficients (*D*_Li_^+^) with increasing the relative contents of poly(S‐*r*‐Ccys) in the Li_2_S/poly(S‐*r*‐Ccys) solution, measured by ^7^Li PFG NMR experiment (Figure 3C). The gradual reduction in *D*_Li_^+^ with the addition of poly(S‐*r*‐Ccys) in Li_2_S/poly(S‐*r*‐Ccys) solution can be attributed to the restriction of Li^+^ ion motion induced by increased Li^+^ coordination.

Density functional theory (DFT) calculations elucidate the plausible interactions between Li_2_S and organo‐polysulfide for a comprehensive understanding of the function of poly(S‐*r*‐Ccys) in stimulating Li_2_S solubility in the ether electrolyte. Dissolution mechanisms can be attributed to an energy‐driven complexation process where molecules adopt specific geometries possessing minimal potential energy [49]. The Li─S bond length in the optimized structure of an isolated Li_2_S unit is estimated to be 2.08 Å (Figure 3D), which matches well with the literature [47]. However, the Li─S bond is observed to be significantly elongated from 2.08 to 2.31 Å and 2.14 Å after electrostatic interactions with oxygen (S─Li*^δ^* ^+^─O*^δ^* ^–^) and nitrogen (S─Li*^δ^* ^+^─N*^δ^* ^−^), respectively. Simultaneously, an increase in the bond lengths of C─O and C─N were observed. Furthermore, the Li─N bond length is 2.02 Å, whereas the Li─O bond length is slightly longer at 2.12 Å, suggesting a potentially stronger interaction with the N of the copolymer. To examine this, single‐point energy calculations were performed using the same level of theory on the optimized geometries. The interaction energies (Δ*E*_interaction_) were calculated using the equation in Note S1, and Li─O and Li─N revealed a value of 21.80 and 26.69 Kcal/mol, respectively. This confirms that both the shorter bond distance and the more negative interaction energy in the Li─N complex indicate that Li^+^ preferentially coordinates with the nitrogen of the poly(S‐*r*‐Ccys) copolymer. Therefore, DFT results reveal that organo‐polysulfide can profoundly assist the dissociation and dissolution of Li_2_S in the ether electrolyte.

### 2.3 Electrochemical Performance of Li‐S Batteries

**FIGURE 4:**
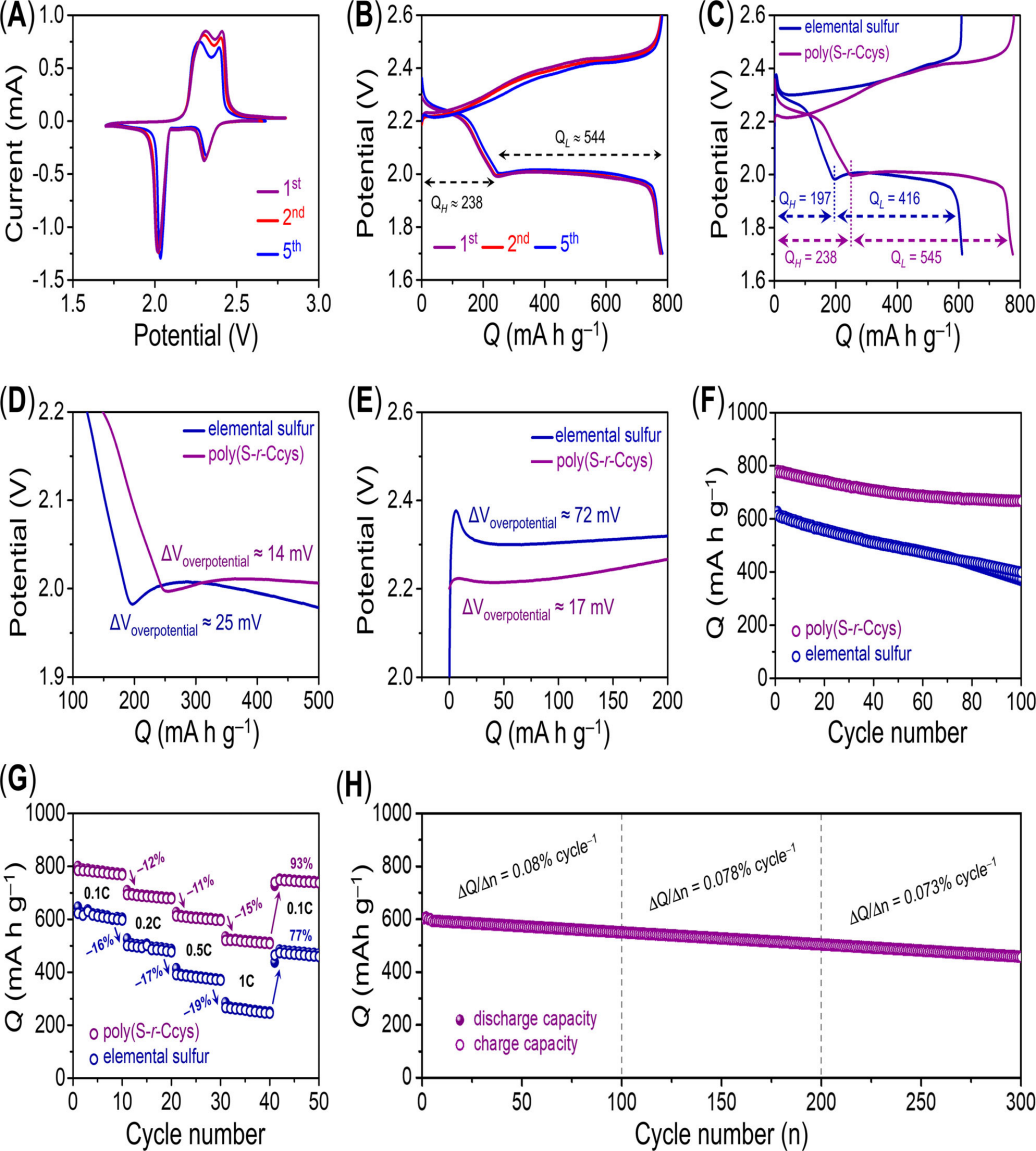
(A, B) Cyclic voltammograms at 0.05 mV s^‒1^ (A) and galvanostatic charge–discharge profiles at 0.1 C (B) of poly(S‐*r*‐Ccys) cathodes, (C) initial cycle of charge–discharge profiles of S_8_ cathode and poly(S‐*r*‐Ccys) cathode at 0.1 C, (D, E) comparison of Li_2_S nucleation overpotential and Li_2_S oxidation overpotential experienced by S_8_ cathode and poly(S‐*r*‐Ccys) cathode, (F) cycling performance comparison at 0.1 C, (G) rate capability comparison, (H) long‐term cycling stability of the poly(S‐*r*‐Ccys) cathode at 1 C.

To evaluate the redox mechanisms of poly(S‐*r*‐Ccys) cathode, cyclic voltammetry (CV) tests were performed at a scan rate of 0.05 mV s^−1^ (Figure 4A). The cyclic voltammograms of the poly(S‐*r*‐Ccys) cathode display two cathodic peaks at 2.28 and 2.02 V (vs. Li^+^/Li), corresponding to a two‐step reduction of poly(S‐*r*‐Ccys) to the end‐discharge product via higher‐order and middle‐order (organo)polysulfides. The poly(S‐*r*‐Ccys) cathode exhibits two anodic peaks, indicating a distinct oxidation mechanism in contrast to the regular oxidation process followed by elemental sulfur (S_8_). A similar two anodic peaks were also observed in our previously reported eugenol‐derived sulfur copolymer cathode [65]. The partial mismatch between the oxidation peaks across successive CV cycles can be attributed to the gradual evolution of the electrode–electrolyte interface and the redistribution of polysulfide species during the initial cycles. Specifically, in the case of the poly(S‐*r*‐Ccys) cathode, the presence of functional groups facilitates strong interactions with lithium polysulfides. This interaction leads to a dynamic redox environment where intermediate sulfur species are progressively immobilized and reorganized. Consequently, the spatial distribution and local concentration of electroactive species may vary slightly between cycles, particularly during the early activation stages of the electrode. Moreover, the morphology of Li_2_S deposition, which is known to impact charge‐transfer kinetics, could be evolving during the initial cycles, leading to subtle shifts in the oxidation peak positions and intensities.

The galvanostatic charge–discharge profiles of the poly(S‐*r*‐Ccys) cathode at 0.1 C are represented in Figure 4B. The discharge profiles reveal two plateaus at 2.28 and 2.02 V, corresponding to the two reduction peaks observed during the cathodic scan in the CV test. Similarly, the charge profiles show two plateaus, analogous to the two oxidation peaks in the cyclic voltammograms. Figure 4C represents a comparative analysis of characteristic charge–discharge profiles of the poly(S‐*r*‐Ccys) and S_8_ cathodes. The S_8_ cathode shows two discharge plateaus, corresponding to the two‐step reduction to Li_2_S. The typical single charge plateau represents the one‐step oxidation of Li_2_S to S_8_ [3]. The pronounced voltage hysteresis observed in the S_8_ cathode reflects sluggish electrochemical kinetics. However, the higher specific capacities observed during both upper‐plateau and lower‐plateau discharge processes of the poly(S‐*r*‐Ccys) cathode suggest enhanced retention of electrolyte‐soluble polysulfides and more efficient conversion of LiPS to Li_2_S [66, 67]. Additionally, the ratio of lower‐plateau discharge capacity (*Q*_L_) to the upper‐plateau discharge capacity (*Q*_H_) serves as a metric for evaluating the conversion efficiency from LiPS to Li_2_S in Li‐S batteries. The poly(S‐*r*‐Ccys) cathode exhibited a *Q*_L_/*Q*_H_ of 2.29, 8.5% higher than that of S_8_ cathode, further corroborating its superior capability in facilitating Li_2_S formation. The slow redox kinetics of the S_8_ cathode were further revealed by the large overpotential during Li_2_S nucleation (25 mV) and Li_2_S oxidation (72 mV), as represented in Figure 4D,E. In contrast, the poly(S‐*r*‐Ccys) cathode shows a lower voltage hysteresis and smaller overpotential during Li_2_S nucleation/oxidation (14 mV and 17 mV, respectively), manifesting the enhancement in redox conversion kinetics of active materials. The significant alleviation of Li_2_S oxidation overpotential (from 72 to 17 mV) could be attributed to the fast reaction kinetics of Ccys‐based organosulfur‐induced enhanced solubility of Li_2_S in the electrolyte. Figure 4F compares the charge–discharge cycling stabilities of the two cathodes. When cycled at 0.1 C, the S_8_ cathode delivered an initial reversible specific capacity of 615 mA h g^‒1^ and retained 372 mA h g^‒1^ after 100 cycles with an average capacity decay rate of 0.39% per cycle. At the same current density, the poly(S‐*r*‐Ccys) cathode exhibited a relatively high initial specific capacity of 782 mA h g^‒1^, implying a higher active material utilization. The poly(S‐*r*‐Ccys) cathode demonstrated excellent cycling stability by retaining 86% of the initial capacity after 100 cycles with an average capacity decay rate of 0.14% per cycle. The poly(S‐*r*‐Ccys) cathode also exhibited superior Coulombic efficiency in comparison to the S_8_ cathode (Figure S9). The CMC binder aids in retaining soluble organo‐polysulfides within the poly(S‐*r*‐Ccys) cathode, as these species tend to bind strongly with polar functional groups [68]. An effective entrapment of soluble organo‐polysulfides can be observed with a higher weight fraction of the CMC binder, as shown in Figure S10. Additionally, to confirm the polysulfide adsorption capability of the Ccys unit in the poly(S‐*r*‐Ccys) copolymer, a polysulfide adsorption experiment was conducted. As shown in Figure S11, the dark coloration of the LiPS solution significantly faded upon introducing the Ccys monomer. The poly(S‐*r*‐Ccys) cathode demonstrated enhanced rate capability and consistently high Coulombic efficiency compared to the S_8_ cathode (Figure 4G and Figure S12). During long‐term cycling at 1 C, the poly(S‐*r*‐Ccys) cathode delivered an initial capacity of 607 mA h g^−1^ and retained 76.7% after 300 cycles, with a low capacity decay rate of just 0.077% per cycle and excellent Coulombic efficiency (Figure 4H and Figure S13). Notably, this stable performance is achieved even at a relatively high active material loading of 4.32 mg cm^−2^, demonstrating an excellent electrochemical performance comparable or superior to the elemental sulfur based cathodes with more complex architectures (Table S1).

**FIGURE 5:**
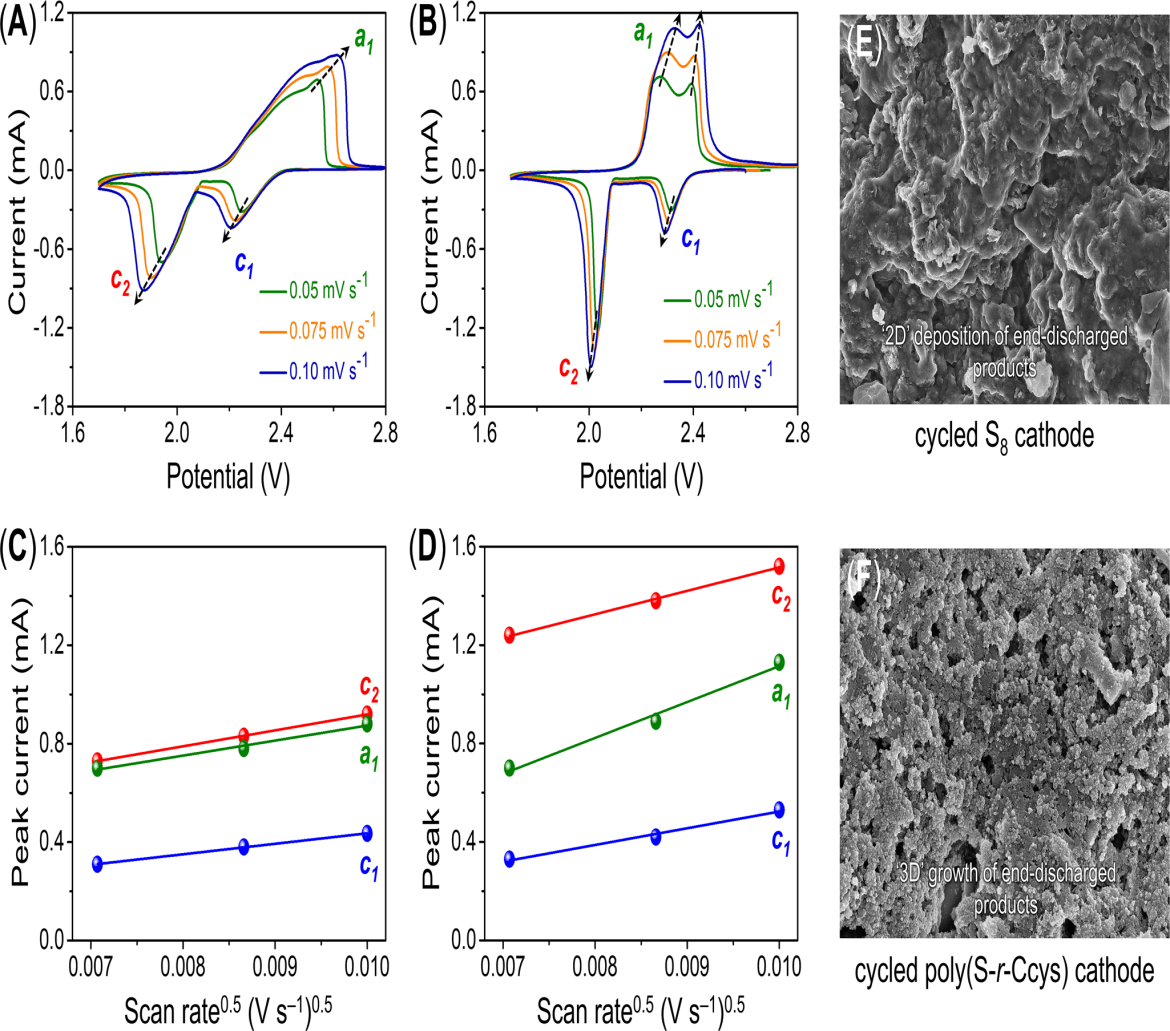
(A, B) Cyclic voltammograms of the S_8_ cathode (A) and poly(S‐*r*‐Ccys) cathode (B) at various scan rates, (C, D) linear fitting of the square root of scan rate and peak current for the S_8_ cathode (C) and poly(S‐*r*‐Ccys) cathode (D), (E) SEM image of the S_8_ cathode at fully discharged state, (F) SEM image of the poly(S‐*r*‐Ccys) cathode at fully discharged state.

The kinetics of Li^+^ diffusion in the poly(S‐*r*‐Ccys) cathode were evaluated using scan rate‐dependent CV tests and compared with that of the S_8_ cathode. The CV profiles of the S_8_ cathode exhibit two cathodic peaks (c_1_ and c_2_) and an anodic peak (a_1_). The pair of cathodic peaks corresponds to a two‐step reduction of S_8_ to Li_2_S transitioning through polysulfide intermediates. The anodic peak is ascribed to the reverse mechanism (Figure 5A,B). The broad redox peaks for the S_8_ cathode suggest sluggish electrochemical kinetics. However, the poly(S‐*r*‐Ccys) cathode displayed relatively sharp redox peaks with higher current density, divulging superior redox kinetics and higher utilization of active materials. The peak current densities and the extent of the negative/positive shift of the cathodic/anodic peaks were enhanced simultaneously with increasing the magnitude of the scan rates for both the cathodes (Figure 5C,D). According to the Randles−Sevcik equation (see Note S2), the linear relationship between the peak currents and the square root of the scan rates reveals the diffusion‐controlled redox processes in the electrodes [23]. As listed in Table S2, the Li^+^ ion diffusion coefficients (*D*_Li_^+^) in the S_8_ cathode were estimated to be 2.47 × 10^−9^, 5.68 × 10^−9^, and 5.05 × 10^−9^ cm^2^ s^−1^ for c_1_, c_2_, and a_1_, respectively. However, the poly(S‐*r*‐Ccys) cathode demonstrated higher *D*_Li_^+^ values of 6.24 × 10^−9^, 1.23 × 10^−8^, and 2.89 × 10^−8^ cm^2^ s^−1^ for c_1_, c_2_, and a_1_, respectively, corroborating accelerated redox kinetics. Moreover, a poly(S‐*r*‐Ccys) symmetric cell incorporating Li_2_S_6_ catholyte exhibited four distinct redox peaks (Figure S14), implying an excellent catalytic behavior of the copolymer towards overall active material utilization.

Considering the impact of the nucleation and growth of charge‐insulating Li_2_S (ionic conductivity ∼ 10^−9^ S cm^−1^, electronic conductivity ∼ 10^−13^ S cm^−1^) [69, 70, 71] toward the overall electrochemical performance of Li‐S batteries, the morphologies of the cycled S_8_ and poly(S‐*r*‐Ccys) electrodes were examined before cycling and at the fully discharged state. The SEM image of a fresh S_8_ cathode exhibits an agglomerated morphology with low porosity that could obstruct the percolation of electrolytes into the bulk of the electrode (Figure S15A). However, the SEM image of the poly(S‐*r*‐Ccys) electrode reveals a porous morphology with plenty of open channels, which can facilitate electrolyte percolation (Figure S15B). The S_8_ cathode exhibits a dense, two‐dimensional (2D) layer of Li_2_S after 100 cycles at the fully discharged state (Figure 5E and Figure S16A). Such spontaneous deposition of the ion‐/electron‐insulating Li_2_S film inhibits the mass transport in the electrodes, which causes lower utilization of active material, large voltage hysteresis, and gradual decay in the specific capacity [34]. However, a porous morphology with the three‐dimensional (3D) growth of Li_2_S was observed in the fully discharged poly(S‐*r*‐Ccys) cathode after 100 cycles (Figure 5F and Figure S16B). We ascribe the growth of 3D Li_2_S in the poly(S‐*r*‐Ccys) cathode to the enhanced solubility of Li_2_S in the electrolyte induced by the novel Ccys benzoxazine‐based hetero atoms‐rich organic moiety present in the sulfur copolymer and consequently the slow rate of Li_2_S nucleation during electrochemical discharge. The 3D porous morphology of Li_2_S inevitably renders the steady percolation of Li^+^ ions into the bulk of the electrodes, resulting in higher utilization of active material in the poly(S‐*r*‐Ccys) cathode, smaller voltage hysteresis, and excellent stability [34]. The bulk electronic conductivity of the pristine poly(S‐*r*‐Ccys) pellet was measured to be 1.11 × 10^−4^ S cm^−1^ that increased to 0.18 × 10^−4^ S cm^−1^ upon incorporating 20 wt% super P conducting carbon additive. The EIS spectrum of a cycled S_8_ cathode reveals a semicircle at the mid‐frequency range (Figure S17), confirming the formation of a continuous 2D Li_2_S film on the S_8_ cathode [72]. In contrast, the EIS spectrum of the poly(S‐*r*‐Ccys) cathode does not show this semicircle at the same frequency range (Figure S17), supporting the hypothesis that the 3D porous structure of Li_2_S leads to minimal mass transfer resistance.

**FIGURE 6:**
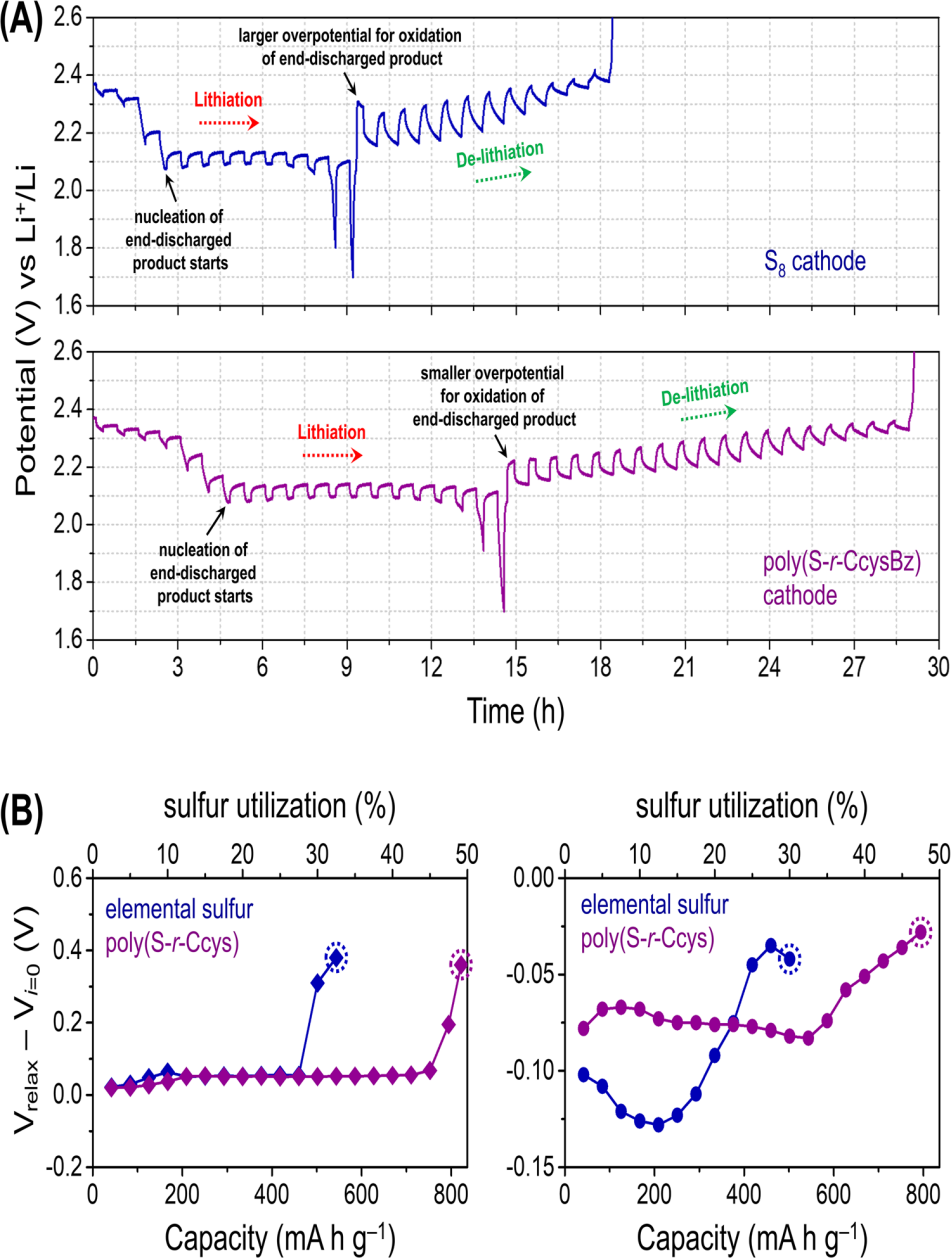
(A) GITT profiles of the S_8_ and poly(S‐*r*‐Ccys) cathodes at 0.1 C, (B) voltage changes after relaxation at different states of discharge (left) and charge (right) for the two cathodes; the dotted circles represent the end of discharging/charging.

The galvanostatic intermittent titration technique (GITT) was employed to perceive the origin of voltage hysteresis for S_8_ and poly(S‐*r*‐Ccys) cathodes in Li‐S batteries (Figure 6A). The overpotential for Li‐S cells containing the S_8_ cathodes increased remarkably upon applying negative current pulses during discharge. Consequently, the cell potential attained the cut‐off voltage of 1.7 V within 10 h. On the contrary, relatively low overpotential was experienced by Li‐S cells containing the poly(S‐*r*‐Ccys) cathodes, and the cells persisted for more than 14 h during the GITT experiment. The magnitudes of voltage increase/decrease during the relaxation period preceded by each negative/positive current pulse for both cathodes at different states of lithiation/de‐lithiation are analyzed in Figure 6B. The magnitude of the voltage‐relaxation is expressed as *V*_relax_—*V_i_*_= 0_, where *V*_relax_ is the equilibrium voltage achieved by the cells after the relaxation period, and *V_i_*_= 0_ is the voltage observed after the current pulses are discontinued. The magnitudes of voltage relaxation were comparable for both cathodes during lithiation in the discharge process. However, a relatively significant voltage relaxation, along with a higher overpotential during the oxidation of the end‐discharge product was observed for S_8_ cathodes. This could be due to inhomogeneity caused by the irregular 2D deposition of the end‐discharged product. In contrast, the smaller magnitudes of voltage relaxation observed for the poly(S‐*r*‐Ccys) cathode during charge further corroborate the homogeneous Li^+^ ion diffusion and superior mass transport due to the porous morphology of the discharged electrode induced by controlled 3D growth of the end‐discharged product.

Considering the observed excellent electrochemical features of the poly(S‐*r*‐Ccys), we aim to evaluate the cycling performance of poly(S‐*r*‐Ccys) cathode considering the practical parameters such as high active material loading (7.8 mg cm^−2^) and under lean‐electrolyte condition (i.e. an electrolyte‐to‐active material ratio of 6.4 µL mg^−1^). When cycled at 0.1 C, the high loaded poly(S‐*r*‐Ccys) cathode delivered an initial specific capacity of 704 mA h g^−1^ (corresponding to an areal capacity of 5.5 mA h cm^−2^) and retained 79% of the initial specific capacity after 50 cycles (Figure S18). The observed cycling results indicate that the poly(S‐*r*‐Ccys) cathode exhibits stable electrochemical performance even under practically relevant conditions. These findings further validate the structural integrity of the cathode design and underscore its applicability in high‐loading and electrolyte‐lean configurations.

Density functional theory (DFT)‐based theoretical calculations were carried out to reveal the superior electrochemical kinetics of the poly(S‐*r*‐Ccys) copolymer at the atomic level. A truncated monomeric model of lithium organo‐polysulfide with one side chain of polysulfide is considered for clarity (Figure S19). A clear distinction between the HOMO‐LUMO band gap of the “lithium organo‐polysulfides” in comparison to pristine “lithium polysulfides” (LiPS based on elemental sulfur) is presented in Figure S20. When the organic unit is present, charges are redistributed along the ‘S’ chain, which reduces the LUMO energy states relative to a LiPS of the same length and gradually shrinks the energy band gap. The redox kinetics is improved as a result of the altered LUMO and HOMO energy levels, which affect the redox capabilities of ‘Lithium organo‐polysulfides.’ Additionally, the Gibbs free energy profile (Figure S21) associated with the discharge process from S_8_ and poly(S‐*r*‐Ccys) to end‐discharge product via lithium polysulfides (for S_8_) and lithium organo‐polysulfides (for copolymer) demonstrates better redox kinetics in poly(S‐*r*‐Ccys) cathode.

**FIGURE 7:**
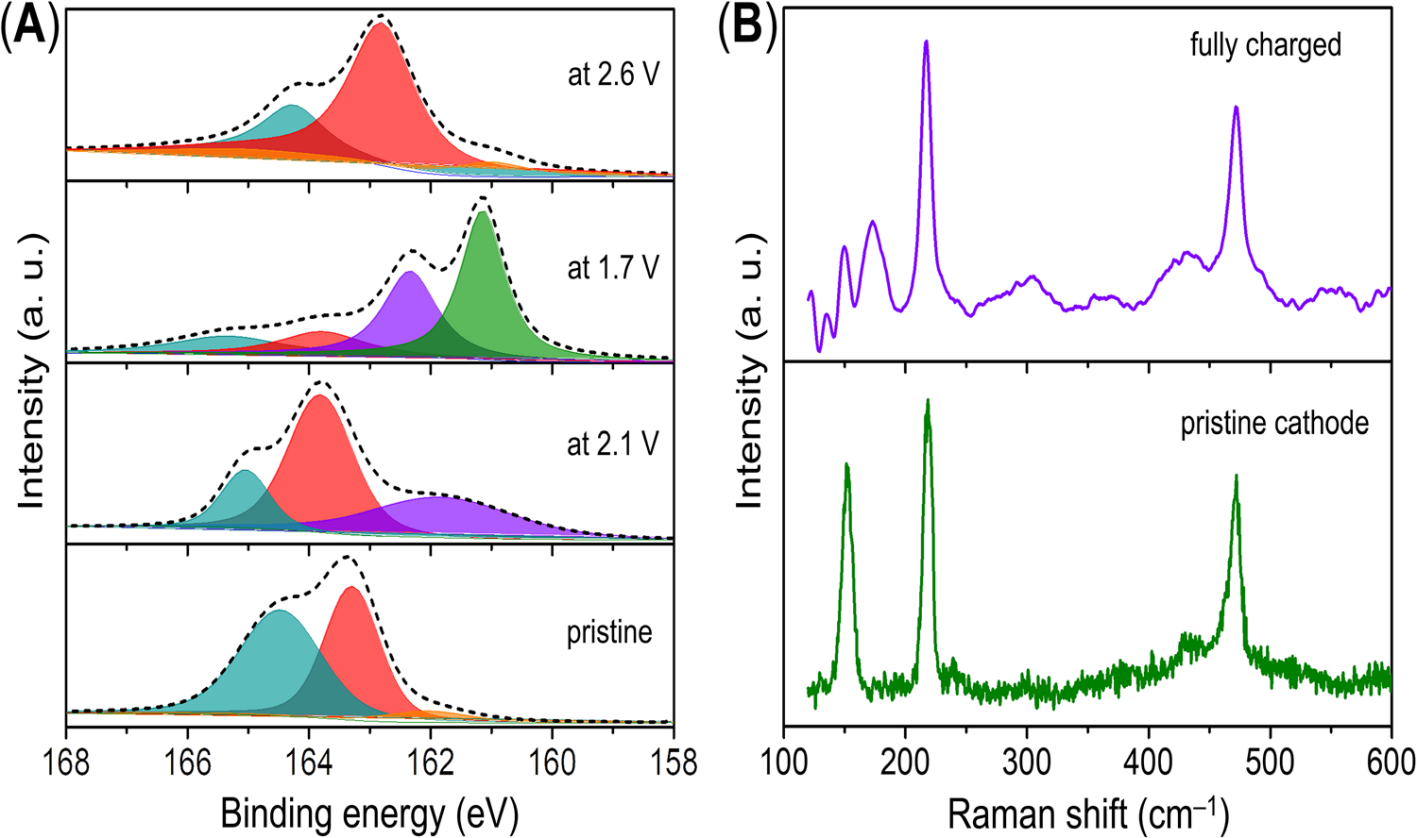
(A) Deconvoluted S2p XPS spectra of poly(S‐*r*‐Ccys) cathodes at various states of discharging and charging, (B) ex‐situ Raman spectra of pristine and fully charged poly(S‐*r*‐Ccys) cathodes.

The electrochemical behavior of the poly(S‐*r*‐Ccys) was investigated by probing the chemical structure evolution in the copolymer at different states of discharge and charge. The deconvoluted S 2p XPS spectrum of pristine copolymer cathode exhibited the characteristic peak components of poly(S‐*r*‐Ccys), as indicated in Figure 7A. A detailed analysis of the S 2p XPS spectrum of poly(S‐*r*‐Ccys) was discussed earlier. The deconvoluted S 2p XPS spectrum of partially discharged poly(S‐*r*‐Ccys) cathode up to 2.1 V bestows an additional peak component (violet‐shaded curve) centered at 161.8 eV that could be ascribed to the formation of middle‐order lithium sulfides (Li_2_S_4_ and Li_2_S_2_) along with lithiated organo‐polysulfides (C–S*_x_*–Li) [17, 73, 74, 75]. However, the intensity of the characteristic S 2p XPS peak components of the poly(S‐*r*‐Ccys) cathode gradually depreciated during the progress of discharge. Eventually, it was disparaged at the end of discharge (i.e. at 1.7 V vs. Li^+^/Li), suggesting a high active material utilization. Moreover, a distinct S 2p XPS peak component (green‐shaded curve) centered at 161.1 eV emerged at 1.7 V. Considering the characteristic binding energy of S 2p electrons in Li_2_S is 160.2 eV [17], we ascribe the peak component observed at the binding energy of 161.1 eV to the strong interaction between ─OH functional groups and S^2−^ (O─H*^δ^* ^+^─S^2–^) via hydrogen bonding. The intense interaction between Li_2_S and the ─OH functional groups in poly(S‐*r*‐Ccys) cathode provides strong binding energy to the S 2p electrons, as observed in the S 2p XPS characterization. This additional hydrogen bonding (O─H*^δ^* ^+^─S^2−^), besides the electrostatic interactions with oxygen (S─Li*^δ^* ^+^─O*^δ^* ^−^) and nitrogen (S─Li^δ+^─N^δ−^), might also promote the dissolution of Li_2_S in the poly(S‐*r*‐Ccys) cathode. Therefore, with the aid of S 2p XPS spectrum analysis, the final discharge products of poly(S‐*r*‐Ccys) cathode can be proposed as a mixed phase of Li_2_S and the lower‐order lithium organopolysulfides where mutual electrostatic interactions exist. Interestingly, the deconvoluted S 2p XPS spectrum obtained from the fully charged poly(S‐*r*‐Ccys) cathode displays only the characteristic peak components of the copolymer, closely resembling the spectrum of the pristine cathode. This observation suggests that the poly(S‐*r*‐Ccys) copolymer could successfully retain its original structure, in which the long‐chain polysulfur units are chemically linked to the carbon atoms in the Ccys moiety, with minimal rearrangement after fully charging the Li‐S batteries. The reversible structural evolution of the poly(S‐*r*‐Ccys) was further supported by ex situ Raman characterization of the copolymer cathode at a fully charged state (Figure 7B). However, the slight change in the appearance of the Raman spectrum of the fully charged poly(S‐*r*‐Ccys) cathode can be ascribed to a certain extent of structural rearrangements in the copolymer during the initial lithiation/de‐lithiation processes.

## 3 Conclusion

In summary, this work demonstrates that regulating the nucleation and growth of insulating Li_2_S is essential for improving the electrochemical performance of Li‐S batteries. We introduce a heteroatom‐rich copolymer, poly(S‐*r*‐Ccys), as a sulfur cathode that enhances Li_2_S solubility and promotes favorable 3D deposition through strong electrostatic interactions with its functional moieties. Enhanced Li^+^ diffusion and redox kinetics were confirmed by Randles–Sevcik analysis, GITT, and PFG‐NMR, while DFT calculations supported the interaction mechanism. Ex situ SEM imaging further validated the controlled Li_2_S growth morphology. Under high‐loading (7.8 mg cm^−2^) and lean‐electrolyte (6.4 µL mg^−1^) conditions, the poly(S‐*r*‐Ccys) cathode delivered an areal capacity of 5.5 mAh cm^−2^ and retained 79% of its capacity over 50 cycles at 0.1 C, highlighting its structural stability and practical viability. This study establishes a promising design strategy for sustainable polymer‐based sulfur cathodes with intrinsic redox‐active functionalities for high‐performance Li‐S batteries.

## 4 Experimental Section

### 4.1 Materials

3‐Pentadecylphenol (saturated cardanol, C), cystamine dihydrochloride (cys), sulfur from Alfa Aesar. Lithium sulfide (Li_2_S), *bis*(trifluoromethane)sulfonimide lithium salt (LiTFSI), lithium nitrate (LiNO_3_), 1,3‐dioxolane (DOL), and 1,2‐dimethoxyethane (DME) from Sigma‐Aldrich. Carboxymethylcellulose sodium salt (CMC), styrene‐butadiene rubber (SBR), and super P carbon from TOB Energy (China). Paraformaldehyde from Fisher Scientific, chloroform, ethyl acetate, and hexane from Finar Limited, sodium sulfate (anhydrous) from Chemlabs, dimethyl sulfoxide (DMSO‐*d_6_*), chloroform CDCl_3_ from Eurisotop, *α*‐cyano‐4‐hydroxycinnamic acid (CHCA) from Bruker, and sodium iodide from Spectrochem. All the reagents were used as received without purification.

### 4.2 Synthesis of Cardanol Cystamine Benzoxazine (Ccys) Monomer

The monomer synthesis was adopted from previously reported literature [76]. Cystamine (cys, 0.016 mol, 2.43 g) was added slowly into a pre‐stirred mixture of cardanol (C, 0.032 mol, 10 g) and paraformaldehyde (0.064 mol, 1.92 g, added 2 g) at 80°C in a mixture of toluene/ethanol (2:1, 45 mL). After adding cystamine, the temperature was raised to 90°C, and the reaction mixture was refluxed for 4 h at 90°C. After completion of the reaction (monitored by thin‐layer chromatography), the reaction mixture was allowed to cool, followed by washing it with aqueous NaOH (1 n, 100 mL) in ethyl acetate (200 mL). The organic layer was collected, washed with distilled water (3 × 100 mL), dried over sodium sulfate, and filtered. Removal of the solvent under vacuum yielded a yellowish‐white solid. The crude product was purified by washing with ethanol, resulting in Ccys as a white solid with an 81% yield. FTIR‐ATR (diamond crystal/ν cm^−1^): 2917, 2850, 2222, 2128, 1984, 1902, 1823, 1620, 1576, 1504, 1467, 1428, 1330, 1237, 1153, 1120, 1105, 1042, 1050, 984, 962, 865, 720, 677, 624, 578; ^1^H NMR (400 MHz, CDCl_3_, *δ* ppm): 0.9 (t, 3H, ─CH_2_─CH_3_), 1.2 (s, 27H, ─(CH_2_)*_n_* ─), 1.6 (q, 2H, ─CH_2_─CH_3_), 2.5 (t, 2H, Ar─CH_2_─), 2.9 (t, 2H, S─CH_2_), 3.1 (t, 2H, N─CH_2_─CH_2_), 4.0 (s, 2H, N─CH_2_─Ar), 4.8 (s, 2H, O─CH_2_─N), 6.6 (s, 1H, Ar─H), 6.7 (d, 1H, Ar─H), 6.84 (d, 1H, Ar─H); ^13^C NMR (100 MHz, CDCl_3_, *δ* ppm): 14.3, 22.8, 29.5, 29.7, 29.7, 29.8, 29.8, 31.5, 32.1, 35.8, 38.0, 50.3, 51.0 (N─CH_2_─Ar), 82.5 (O─CH_2_─N), 116.3, 117.2, 121.1, 127.3, 143.2, 154.0. HR (ESI interface‐positive ions): [M + H + 2H_2_O − 2CH_2_O]^+^ 785.6047 (theoretical) 785.6030 (calculated) (Figure S1).

### 4.3 Synthesis of Poly(S‐*r*‐Ccys) Copolymer

The copolymer was synthesized by following previously reported literature [77]. Elemental sulfur (S_8_, 900 mg) was heated to 160°C in a 15 mL glass vial to form an orange‐brown‐colored viscous melt. 100 mg of Ccys was then added directly to the molten polymerized sulfur under magnetic stirring. The reaction was continued at 185°C for 10 min and then allowed to cool down to room temperature slowly. The solid copolymer was ground to obtain a brown powder.

### 4.4 Fabrication of Electrode and Electrochemical Testing

The poly(S‐*r*‐Ccys) cathode was prepared by mixing 70 wt% of sulfur copolymer, 20 wt% of super P carbon black, 8 wt% of carboxymethylcellulose sodium salt (CMC), and 2 wt% of styrene–butadiene rubber (SBR) into a prerequisite amount of deionized water. The composite of the sulfur copolymer and super P carbon black was first balled‐milled for 30 min to reduce the particle size of poly(S‐*r*‐Ccys) and to obtain a homogeneous blend. The as‐obtained slurry was subsequently blade‐cast onto a carbon‐coated aluminum foil (thickness ≈22 µm) and dried at room temperature for 48 h under vacuum. A reference cathode was also prepared by blade‐casting an aqueous slurry comprising 75 wt% of sulfur (S_8_) powder, 20 wt% of super P carbon black, 3.0 wt% of CMC, and 2.0 wt% of SBR. The respective electrodes were cut into circular disks with a diameter of 16 mm and further dried at 60°C for 24 h under vacuum. The average mass loadings of active materials on the poly(S‐*r*‐Ccys) cathode and S_8_ cathode were estimated to be 4.32 mg cm^−2^ and 4.40 mg cm^−2^, respectively. CR2032‐type coin cells were assembled in an argon‐filled glovebox. Lithium metal was used as both reference and counter electrode. Celgard polypropylene membrane was used as the separator soaked with the electrolyte comprised of 1 m LiTFSI and 0.1 m LiNO_3_ in 1:1 (v/v) mixture 1,3‐dioxolane (DOL) and 1,2‐dimethoxy ethane (DME). The electrolyte amount was fixed to 10 µL per milligram of active material. Cyclic voltammetry (CV) and galvanostatic intermittent titration technique (GITT) tests were conducted on a potentiostat (BioLogic VMP‐3 instrument). The CV tests of Li‐S cells were carried out at different scan rates within the potential window of 1.7–2.8 V (vs. Li^+^/Li). During GITT experiments, the Li‐S cells were negatively polarized (during discharging) or positively polarized (during charging) at 0.1 C (1 C = 1672 mA g^−1^) for 15 min. Each current pulse was followed by a relaxation period of 1 h or Δ*V*/Δ*t* ≤ 10 mV h^−1^, whichever is earlier. The galvanostatic charge–discharge experiments were performed on an Arbin BT‐2000 instrument at various prerequisite current rates. All electrochemical measurements were carried out at 20°C.

### 4.5 Computational Methods and Details

All the density functional theory (DFT) calculations have been performed using the Gaussian 09 package [78]. All structures were optimized using the Lee–Yang–Parr correlation functional (B3LYP) at 6–311G++G(d, p) level [79]. The SMD universal solvation model was utilized to characterize solvation effects in the DME environment [80]. The solvent radius and dielectric constant values were 4.19 Å and 7.2, respectively [27]. The ultimate energy of the system was obtained by further rectifying all energies by adding zero‐point energy (ZPE) for each state. The energies and the shape of molecular orbitals (MOs) have been calculated for the optimized geometry and rendered at an isovalue of 0.05. Other theoretical methods and details are provided in the Supporting Information.

### 4.6 Material Characterizations

The structure of the compounds was verified by proton, carbon, distortion enhancement polarization transfer, and 2D heteronuclear single quantum correlation (^1^H, ^13^C, DEPT) nuclear magnetic resonance (NMR) experiments on Bruker AV400 NMR (400 MHz) FT‐NMR spectrometer using deuterated solvents with tetramethylsilane as internal standard. Solutions for ex‐situ ^7^Li NMR experiments on varying amounts of the copolymer in the electrolyte solvent containing Li_2_S were prepared in an argon‐filled glovebox. Typically, 5 mg of Li_2_S was taken in 1 mL of DME/DOL (1:1 v/v) solvent mixture, and varying amounts of poly(S‐*r*‐Ccys) were added and stirred for 12 h. The prepared samples were transferred to the NMR tubes, and a DMSO‐filled capillary was inserted into the tube. The pulse field gradient (PFG) NMR experiment was performed using a Bruker BBO probe with a *z*‐axis gradient coil. The diffusion coefficient was obtained from ^7^Li NMR spectra measured by a stimulated echo bipolar pulse field gradient (stebpgp1s) program with 1 spoil gradient, and the gradient strength was logarithmically increased in 16 steps from 2% to 95% of its maximum. The data was processed and analyzed using MestReNova (14.2.1) software. Fourier‐transform infrared (FTIR) spectra were recorded on a Nicolet iS20 spectrometer equipped with an interferometer, KBr/Ge‐coated beam splitter, deuterated‐triglycine sulfate (DTGS) detector, and attenuated total reflectance diamond (iD5‐ATR) accessory. Sixty‐four scans were recorded at 4 cm^−1^ resolution within 4000–400 cm^−1^, averaged, and referenced to air. The molecular weight distribution of the copolymer was determined using gel permeation chromatography (GPC), Viscotek Model 305 TDA max fitted with Viscotek modular differential refractive index detector (VE 3580 model). Copolymer sample was eluted through two general mixed columns (T6000M, standard styrene‐divinylbenzene copolymer, 300 × 8 mm, maintained at 35°C) using tetrahydrofuran (THF) at a flow rate of 1 mL min^−1^. The instrument was pre‐calibrated with polystyrene standards, and the data was analyzed using Omnisec software. For measurement, samples were dissolved in tetrahydrofuran solvent (4–5 mg mL^−1^, left overnight) and prefiltered through a polytetrafluoroethylene filter (0.2 µm) to remove insoluble particles before injecting into the instrument. Matrix‐assisted laser desorption ionization time‐of‐flight mass spectrometer (MALDI‐ToF MS, Bruker autoflex Max) of the copolymer was performed using the matrix α‐cyano‐4‐hydroxycinnamic acid (CHCA) and sodium iodide salt.

The polymerization and glass transition temperatures of monomer and copolymer were evaluated by DSC‐3, Star system, and Mettler Toledo. For a dynamic differential scanning calorimetry (DSC) scan, the monomer (1 ± 0.2 mg) was enclosed in a hermetic aluminum pan and heated from 30 to 150°C under a nitrogen gas atmosphere at a 50 mL/min constant flow rate. For sulfur, copolymer, and physical blend, one heat–cool (30 to 150°C and 150 to 30°C) cycle was performed at 10°C min^−1^ under the same nitrogen flow rate. Thermogravimetric analysis (TGA) of monomers was performed using a Mettler Toledo thermogravimetric analyzer with a built‐in gas controller (TGA2 SF/1100) and fitted with an XP1U TGA balance (ultra‐microbalance) under a 50 mL/min flow rate of nitrogen in the temperature range of 35−800°C at a heating rate of 10°C min^−1^. Powder X‐ray diffraction (PXRD) was performed on Bruker D8‐Discover using room temperature with Cu‐K radiation (*λ* = 0.154 nm). The average crystallite size (*L*, nm) was calculated using the Debye–Scherrer equation as follows:

L≈λ/βcosθ

where *β* is the full width at half‐maximum (FWHM) of the peak in radians and *θ* is half of the scattering angle 2*θ*. We have utilized 222 reflections (at the highest diffraction peak for the orthorhombic sulfur structure) for the calculation at 2*θ* ∼ 23°. The bulk electronic conductivity was evaluated using a pressed pellet at 0.5 V with a Keithley SCS‐4200 Parameter Analyzer connected to a four‐point probe station with tungsten tips. Two pellets were prepared for the measurements: one made from the pristine copolymer and the other from a copolymer/super P composite (80/20 w/w). Each pellet was formed into circular discs with a thickness of 0.6 mm and a diameter of 5 mm by applying a pressure of 5 tons for 1 min. A micro‐Raman spectrometer (Renishaw inVia) was used to get the Raman spectra. For optical microscopy characterization, a small amount of the sample was placed between the glass slide and a cover slip and heated from 30 to 110°C at a rate of 10°C min^‒1^ and 110 to 150°C at a rate of 2°C min^−1^ and again cooled back to 30°C at a rate of 20°C min^‒1^ using hot stage (Linkam, LTS120, UK) attached to a polarizing microscope (Nikon Eclipse 100N POL, Japan). Images were captured to monitor the phase change in the material during heating. X‐ray photoelectron spectroscopy (XPS) measurements were carried out using a Thermo Fisher NEXSA surface analyzer with a monochromatized Al‐K_α_ (1486.7 eV) radiation source to determine the chemical composition and binding energy. The acquired spectrum was processed using CasaXPS software. The morphologies of discharged cathodes were examined by field emission gun scanning electron microscope (Zeiss, Ultra S5).

## Author Contributions

BL conceived the idea and supervised this work. SM, DYW, AG, supervised the electrochemical work. SS and AG proposed the concept, designed the experiments, and wrote the paper. SAS carried out the DFT calculations. MM, MK, and ST assisted in the experimental work. All authors analyzed and discussed the experimental data. SS, AG, SM, and BL formatted and revised the paper with inputs from all the authors.

## Conflicts of Interest

The authors declare no conflicts of interest.

## Supporting information




**Supporting File**: exp270162‐sup‐0001‐SuppMat.docx.

## Data Availability

All data supporting the findings of this study are included in the article and the Supplementary Information.
