# Supplementary material for: Sulfur‐Rich Sustainable Copolymers for Enhancing Redox Kinetics and Alleviating Cathode Passivation in Lithium‐Sulfur Batteries
Source: Exploration (Beijing). 2026 May 26;6(3):20240447. doi: 10.1002/EXP.20240447 (PMC13317702; doi:10.1002/EXP.20240447)
Supplement: Supplementary file 1 — Supporting File: exp270162‐sup‐0001‐SuppMat.docx. [file EXP2-6-20240447-s001.docx]

Supporting Information

**Sulfur-rich sustainable copolymers for enhancing redox kinetics and alleviating cathode passivation in lithium-sulfur batteries**

Sangeeta Sahu, Arnab Ghosh,* Monisha Monisha, Murali Krishna, Shakir Ali Siddiqui, Sunan Tian, De-Yi Wang,* Sagar Mitra,* and Bimlesh Lochab*

S. Sahu, M. Monisha, S. A. Siddiqui, A. Ghosh, B. Lochab

Department of Chemistry

School of Natural Sciences

Shiv Nadar Institution of Eminence

Gautam Buddha Nagar, Uttar Pradesh, 201314, India.

E-mail: [bimlesh.lochab@snu.edu.in](mailto:bimlesh.lochab@snu.edu.in), [arnab.ghosh@snu.edu.in](mailto:arnab.ghosh@snu.edu.in)

M. Krishna, S. Mitra

Department of Energy Science and Engineering

Indian Institute of Technology Bombay

Powai, Mumbai, Maharashtra 400076, India

E-mail: [sagar.mitra@iitb.ac.in](mailto:sagar.mitra@iitb.ac.in)

A. Ghosh, S. Tian, D.-Y. Wang

IMDEA Materials Institute

C/Eric Kandel, 2, 28906, Getafe, Madrid, Spain.

E-mail: [deyi.wang@imdea.org](mailto:deyi.wang@imdea.org), [arnab.ghosh@imdea.org](mailto:arnab.ghosh@imdea.org)


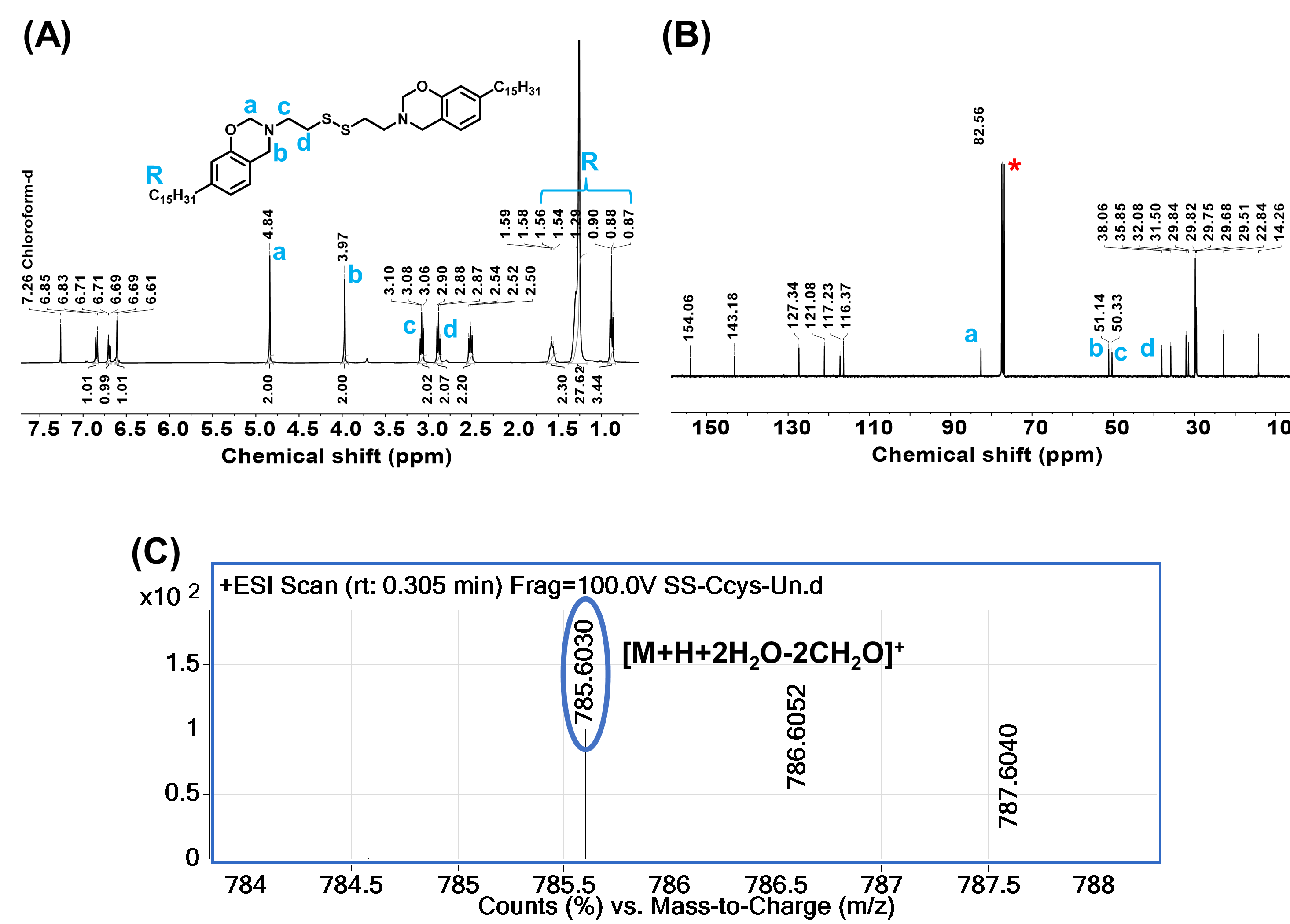


Figure S1. (A) ^1^H NMR spectrum, (B) ^13^C NMR spectrum (recorded in CDCl_3_), and (C) mass chromatograph of Ccys benzoxazine monomer.


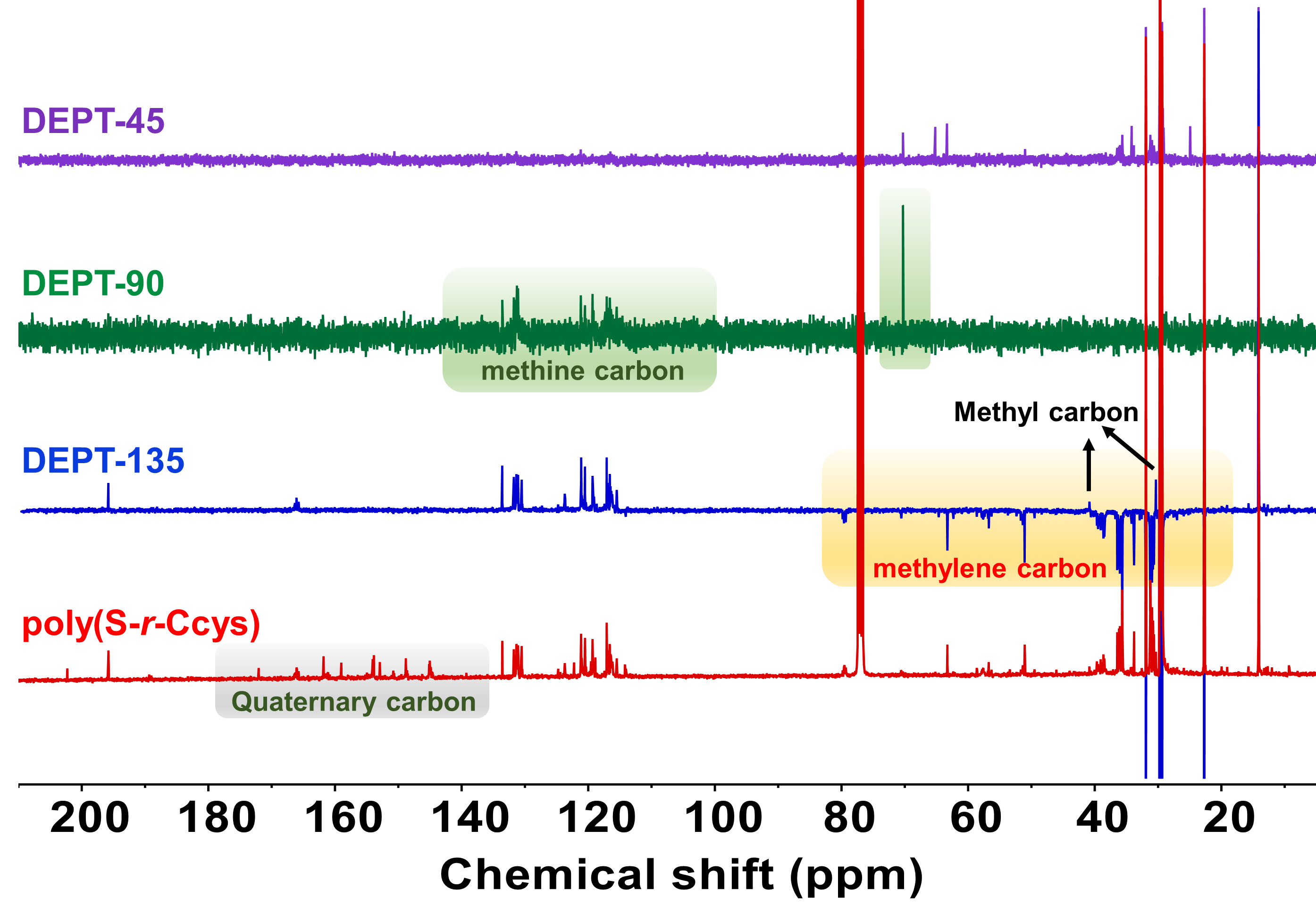


Figure S2. Stacked ^13^C DEPT NMR spectra of poly(S-*r*-Ccys) copolymer recorded in CDCl_3_.


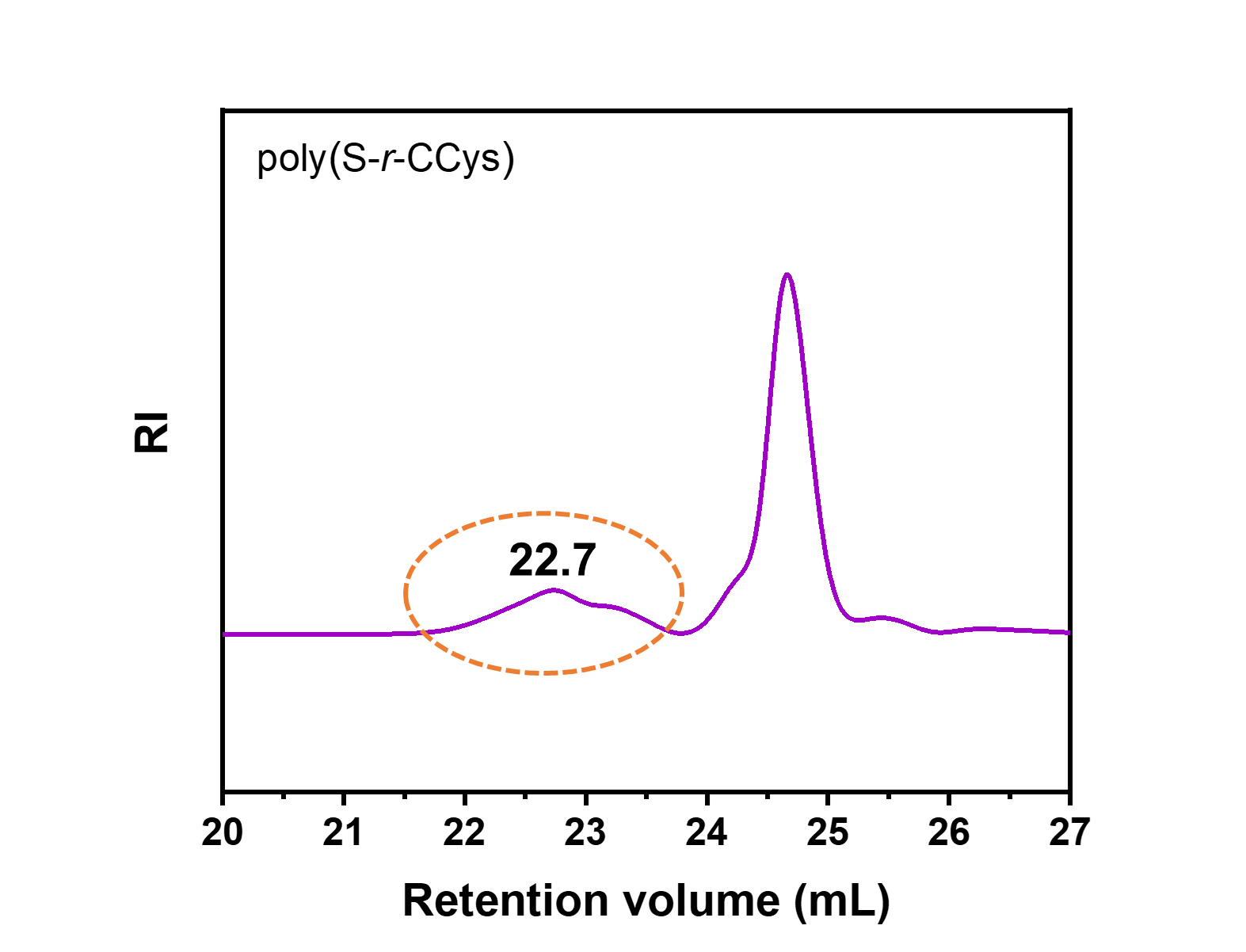


**Figure S3.** GPC trace of the copolymer.


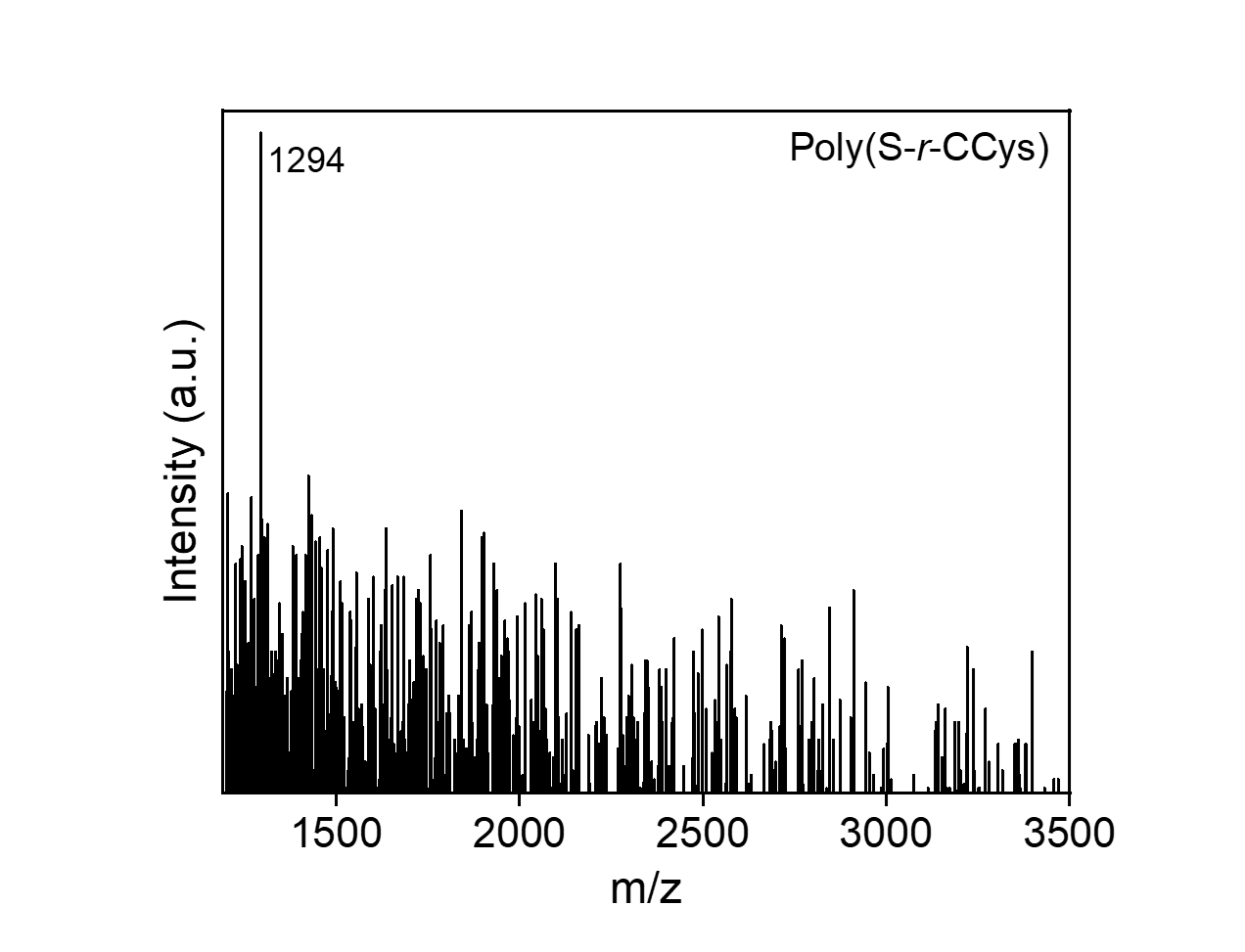


**Figure S4.** MALDI-TOF spectrum of the copolymer.


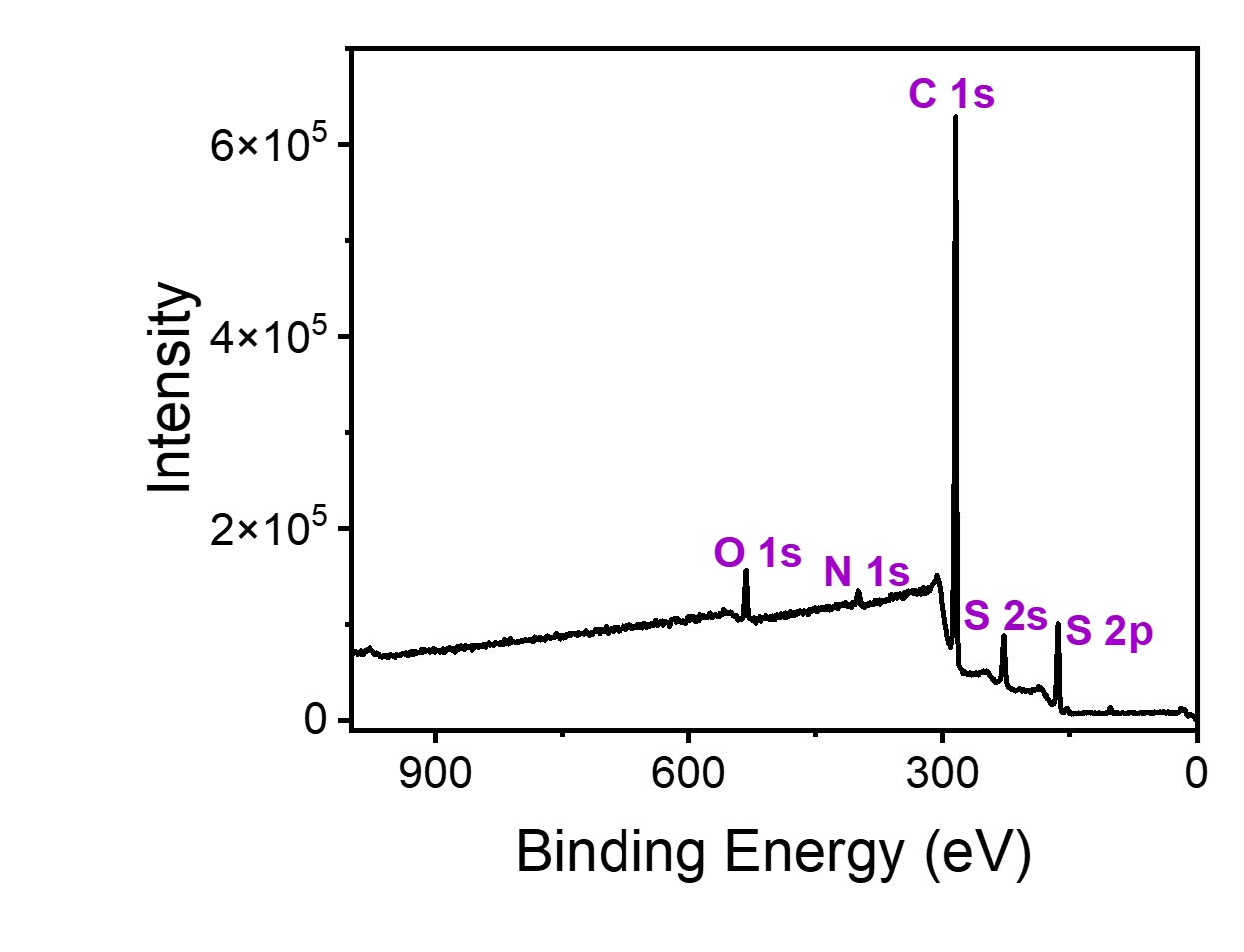


Figure S5. XPS wide-survey scan of poly(S-*r*-Ccys).


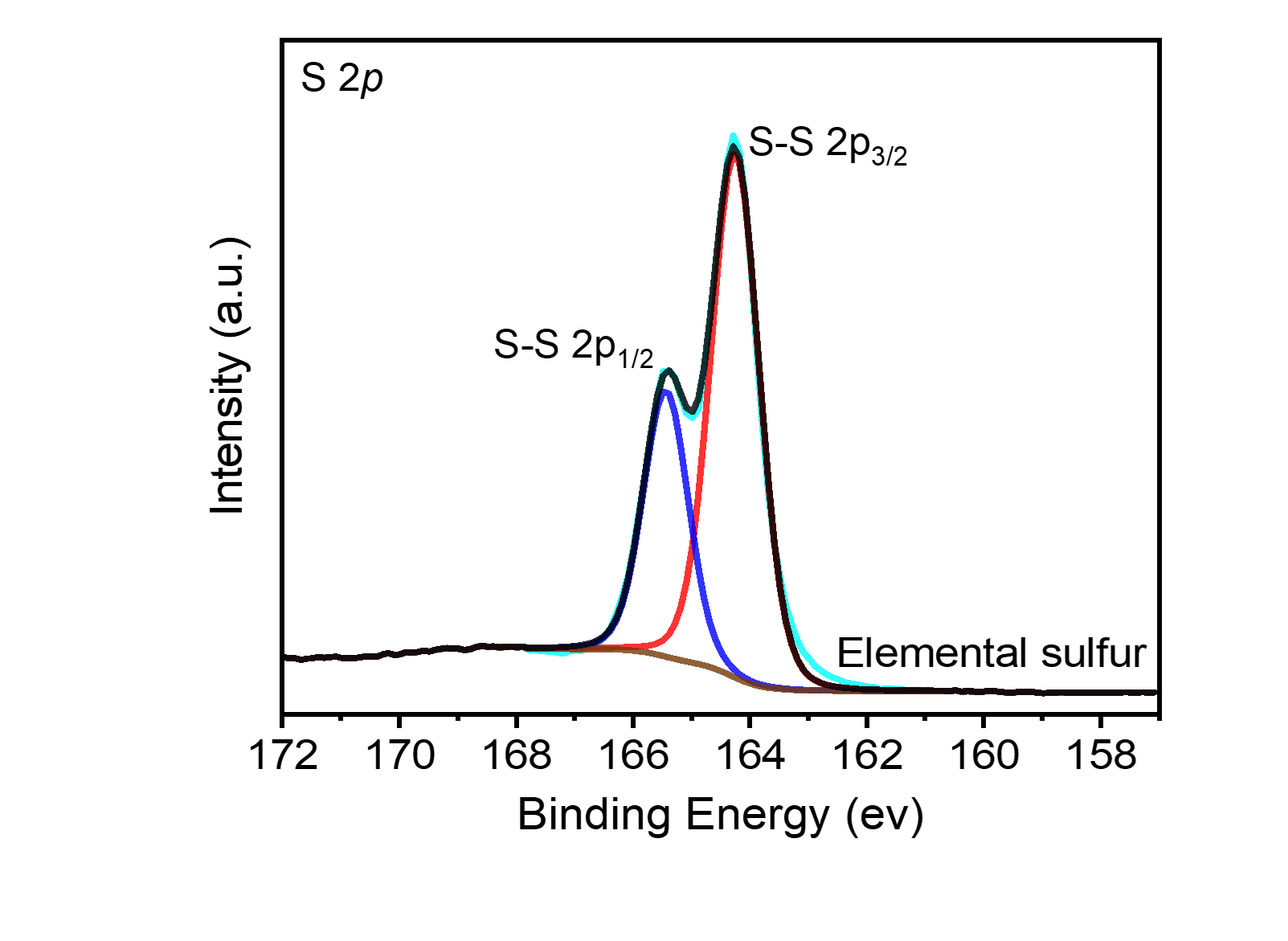


Figure S6. Deconvoluted S 2p XPS spectrum of elemental sulfur.


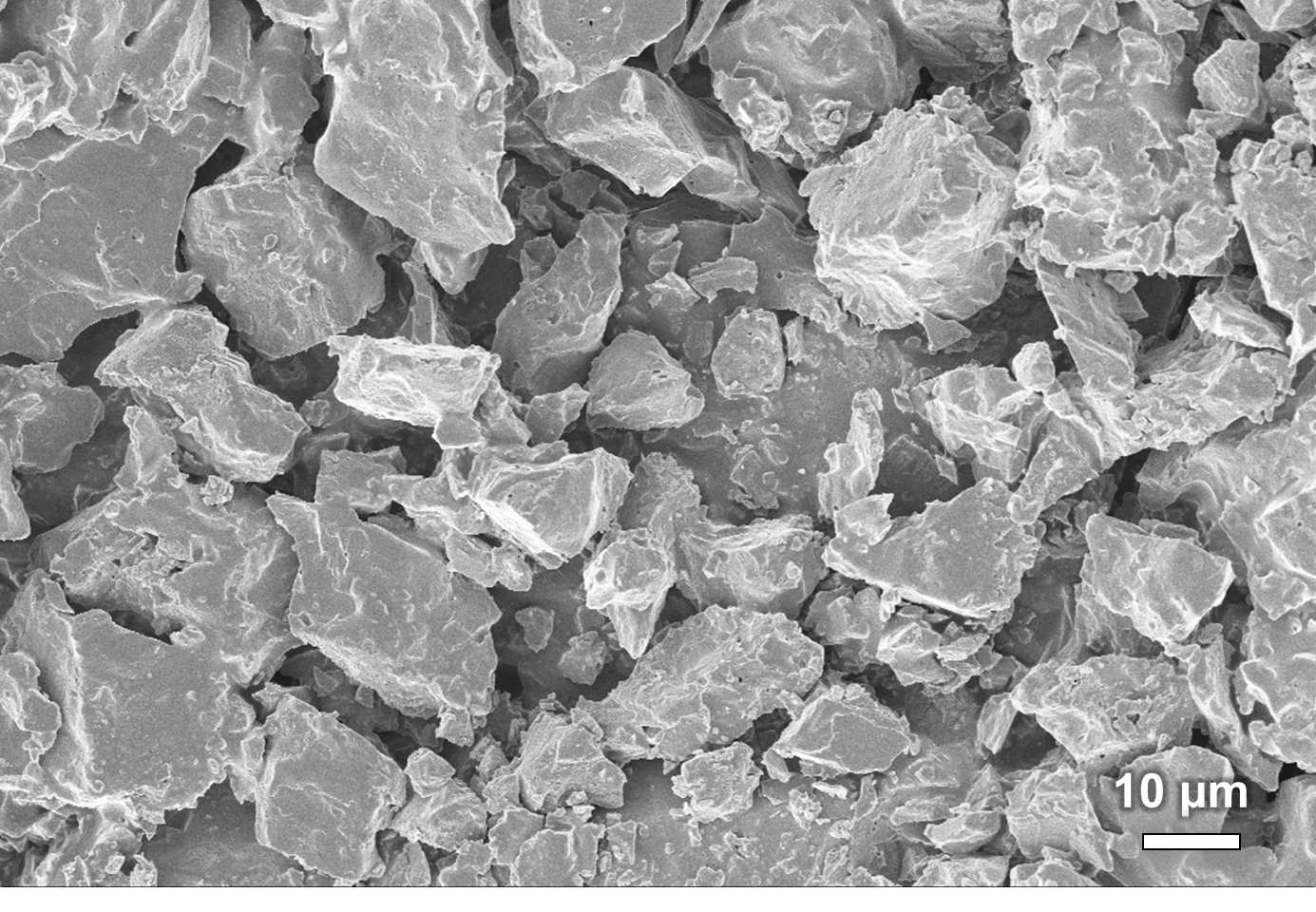


Figure S7. SEM image of poly(S-*r*-Ccys) copolymer.


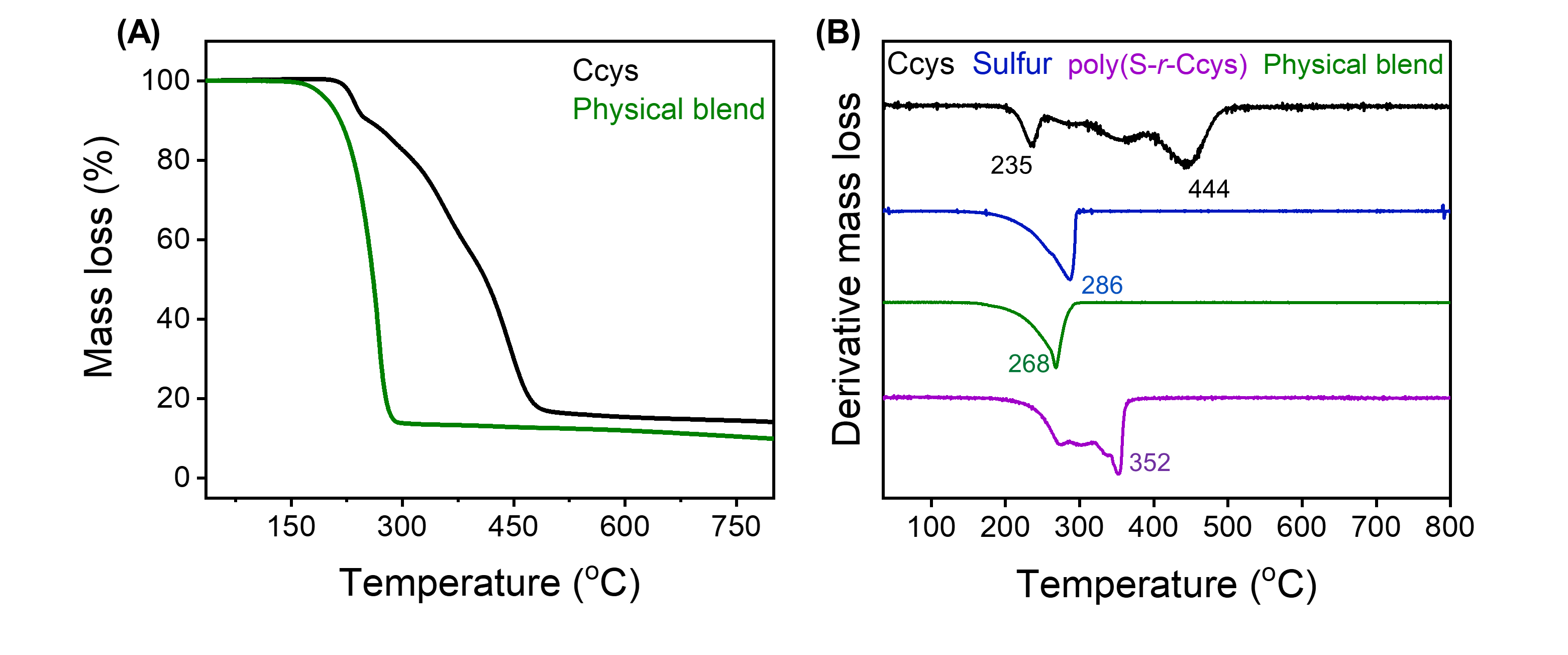


Figure S8. (A) TGA thermogram of Ccys and Ccys:sulfur (1:9) physical blend, (B) Stacked derivative mass loss (DTG) plot of Ccys, sulfur, physical blend, and copolymer.


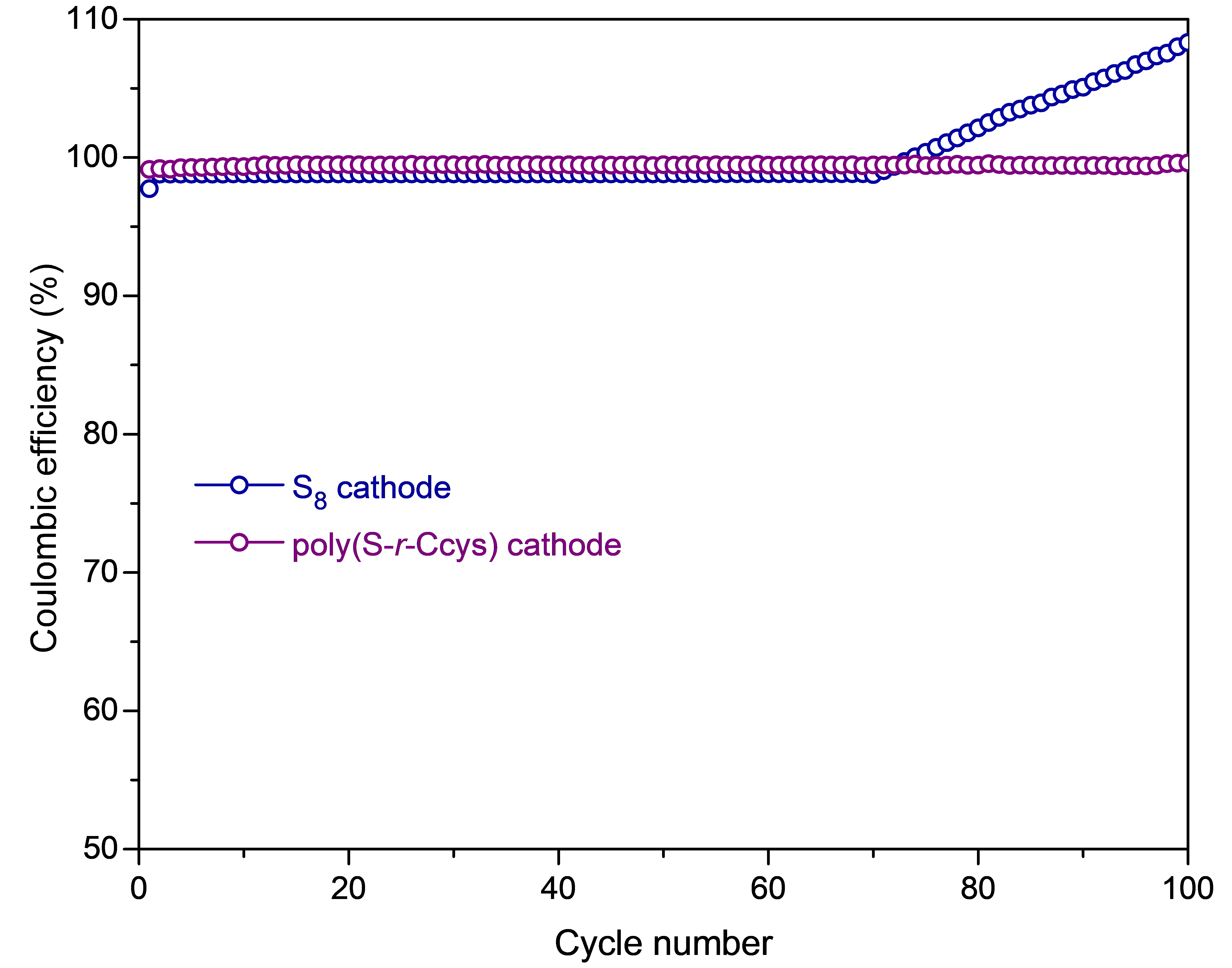


**Figure S9.** Coulombic efficiencies of S_8_ and poly(S-*r*-Ccys) cathodes when cycled at 0.1C.

**
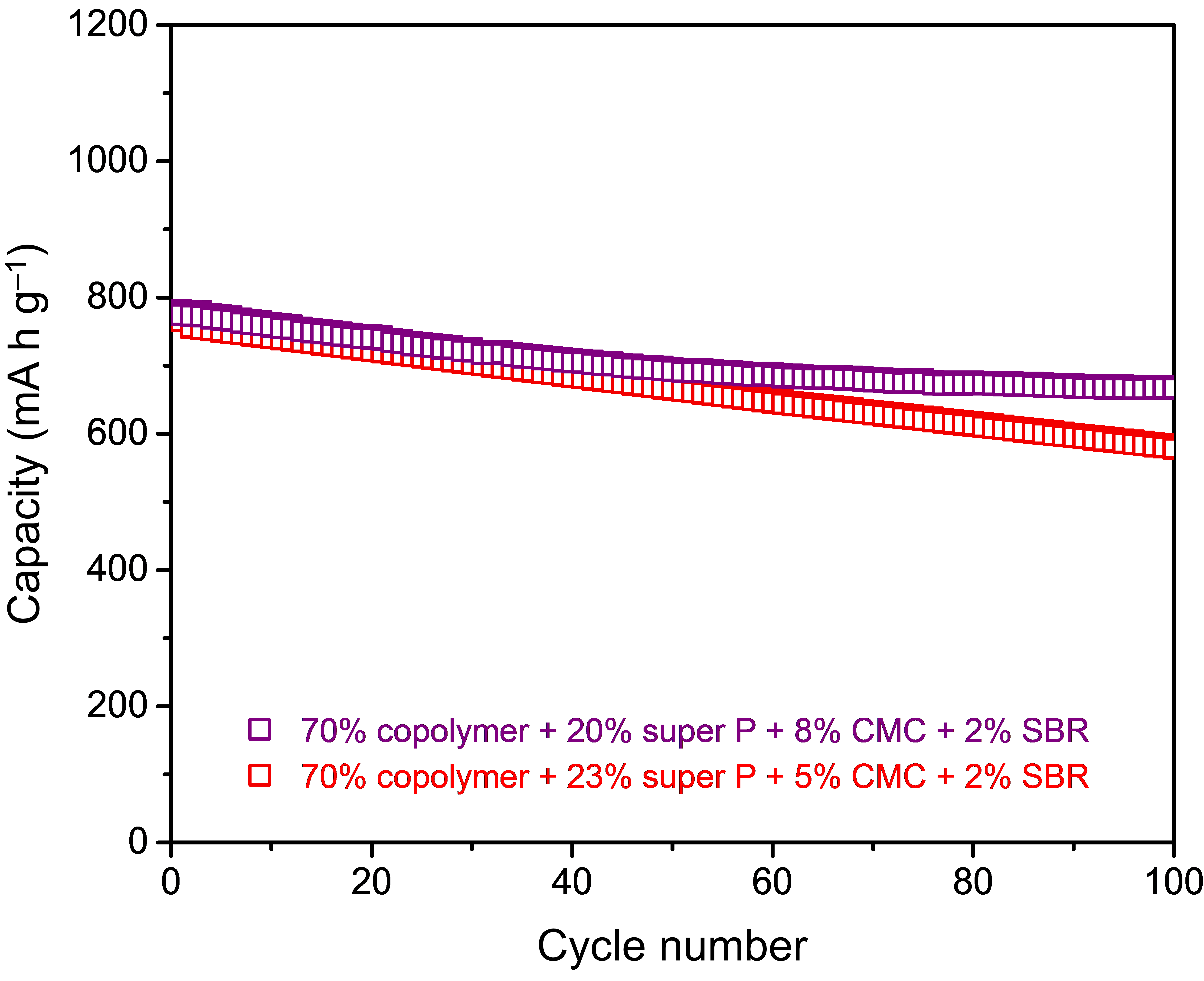
**

**Figure S10.** Charge-discharge cycling stability of different poly(S-*r*-Ccys) cathodes at 0.1C.


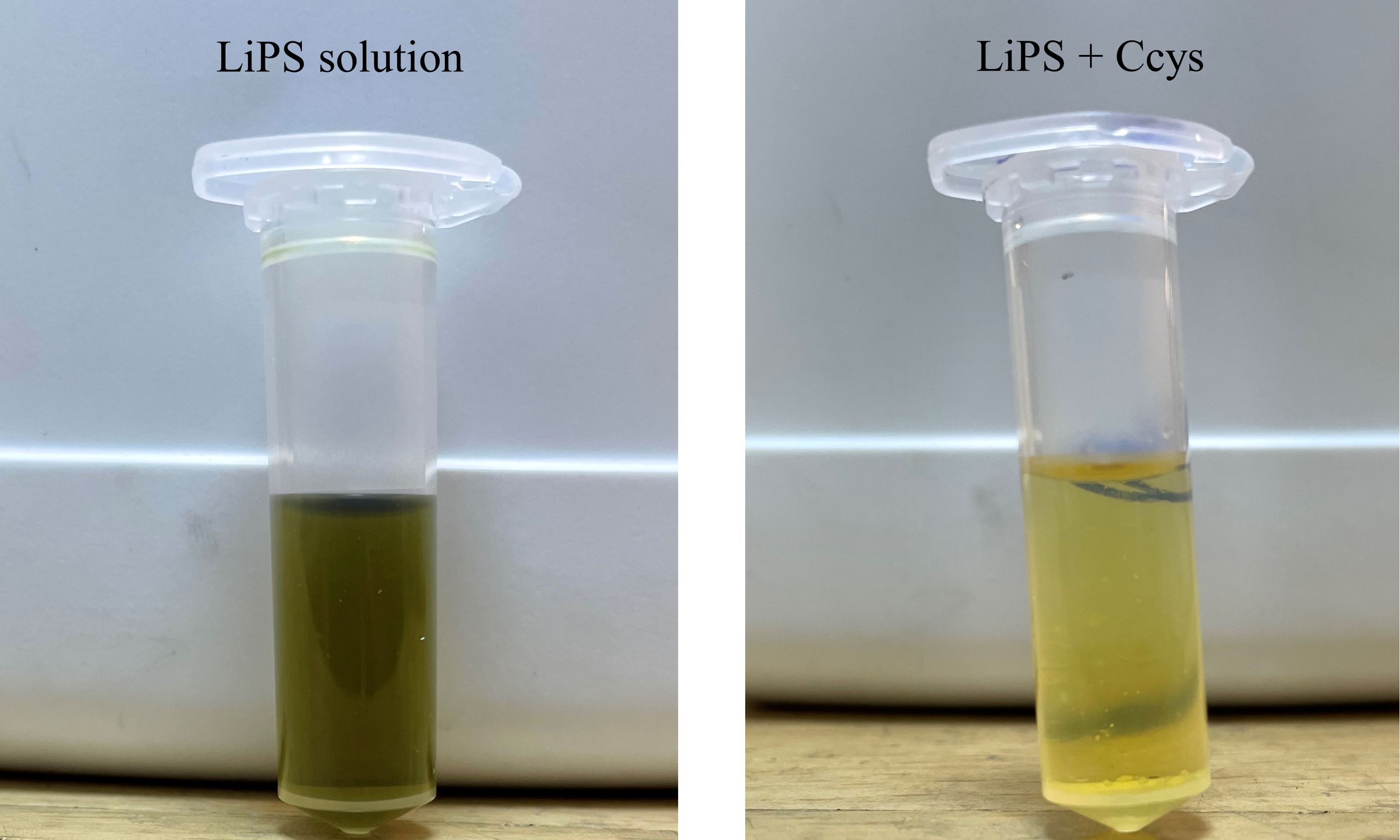


**Figure S11.** Lithium polysulfide solution before and after incorporation of Ccys monomer.

**
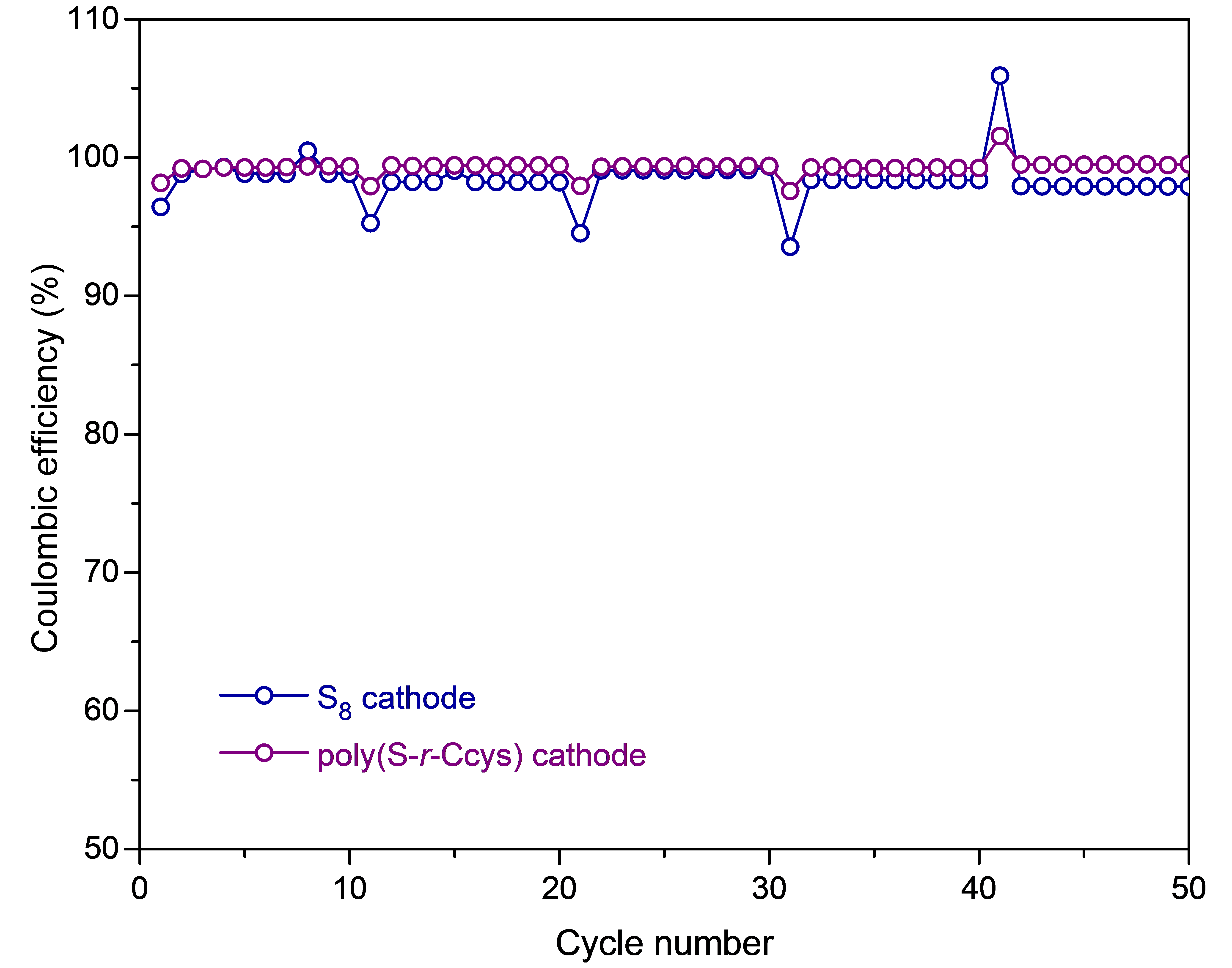
**

**Figure S12.** Coulombic efficiencies of S_8_ and poly(S-*r*-Ccys) cathodes when cycled at various current rates.

**
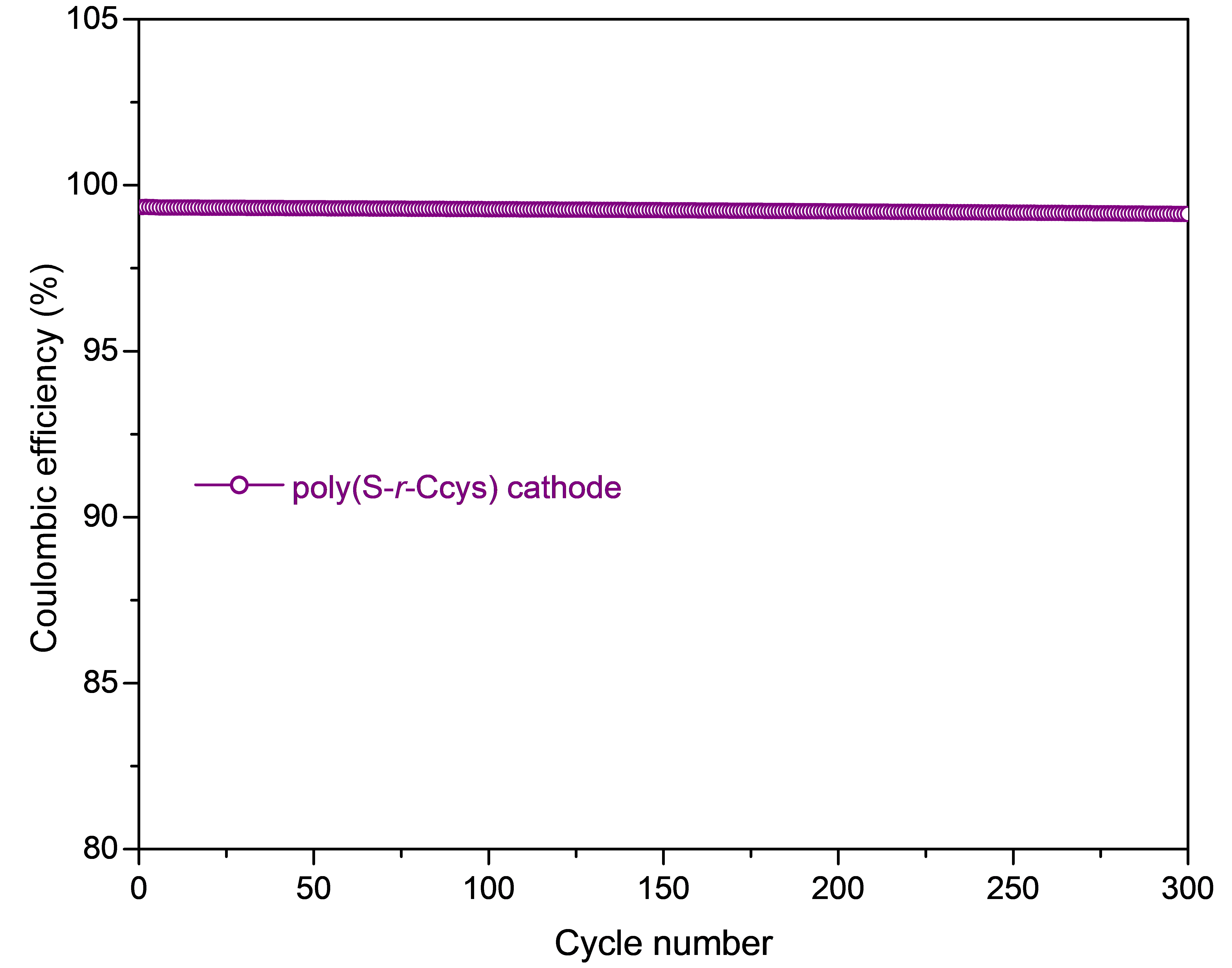
**

**Figure S13.** Coulombic efficiency of poly(S-*r*-Ccys) cathode during the long-term cycling performance at 1C.

Table S1. Electrochemical cycling performance comparison of poly(S-*r*-Ccys) cathode with representative advanced sulfur cathodes

| **Cathode** | **Sulfur loading** | **Initial capacity** | **Rate** | **Retention after 300 cycles** | **Maximum no. of cycles** |
| --- | --- | --- | --- | --- | --- |
| Poly(S-*r*-Ccys)^this work^ | 4.3 mg cm^−2^ | 607 mA h g^−1^ | 1C | 76.7% | 300 |
| Ti_3_C_2_T*_x_*/S_8_^[1]^ | 2.0 mg cm^−2^ | 1225 mA h g^−1^ | 0.5C | 57.5% | 300 |
| MgO/S_8_^[2]^ | 1.1 mg cm^−2^ | 1105 mA h g^−1^ | 0.5C | 83.7% | 300 |
| Al_2_O_3_/S_8_^[2]^ | 1.1 mg cm^−2^ | 1117 mA h g^−1^ | 0.5C | 45.4% | 300 |
| CaO/S_8_^[2]^ | 1.1 mg cm^−2^ | 1152 mA h g^−1^ | 0.5C | 52.1% | 300 |
| Diphenyl diselenide/S_8_^[3]^ | 1.2 mg cm^−2^ | 1056 mA h g^−1^ | 0.5C | 68.2% | 350 |

Table S2. *D*_Li_^+^ values of the S_8_ and Poly(S-*r*-Ccys) cathodes for different electrochemical steps were obtained using the Randles–Sevcik equation.

| **Cathode** | ***D*_Li_^+^** (c_1_) | ***D*_Li_^+^** (c_2_) | ***D*_Li_^+^** (a_1_) |
| --- | --- | --- | --- |
| S_8_ | 2.47 × 10^–9^ cm^2^/s | 5.68 × 10^–9^ cm^2^/s | 5.05 × 10^–9^ cm^2^/s |
| Poly(S-*r*-CcysBz) | 6.24 × 10^–9^ cm^2^/s | 1.23 × 10^–8^ cm^2^/s | 2.89 × 10^–8^ cm^2^/s |

**
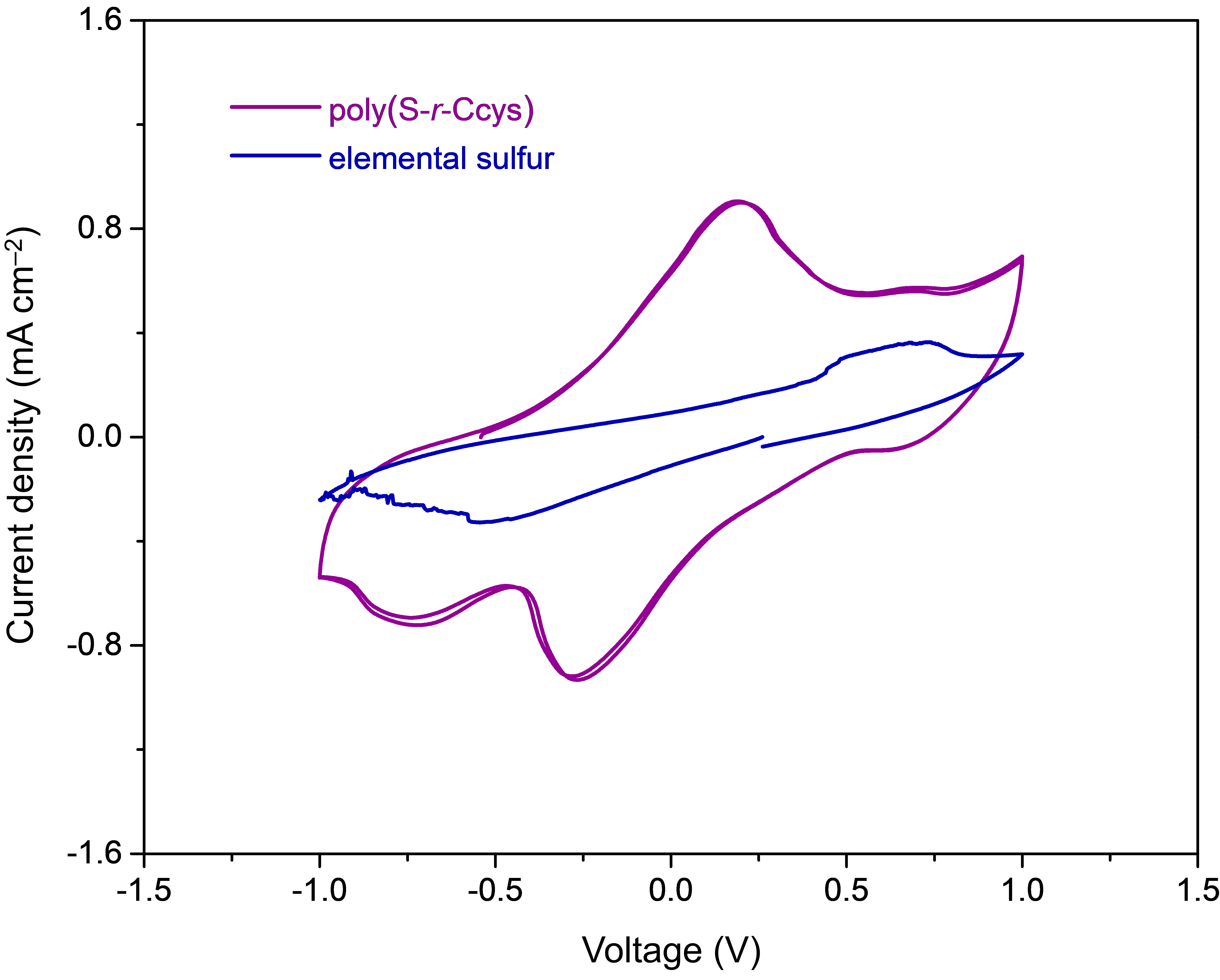
**

**Figure S14.** CV curves of Li_2_S_6_ symmetric cells with poly(S-*r*-Ccys) or S_8_ electrodes at a scan rate of 0.5 mV s^−1^.


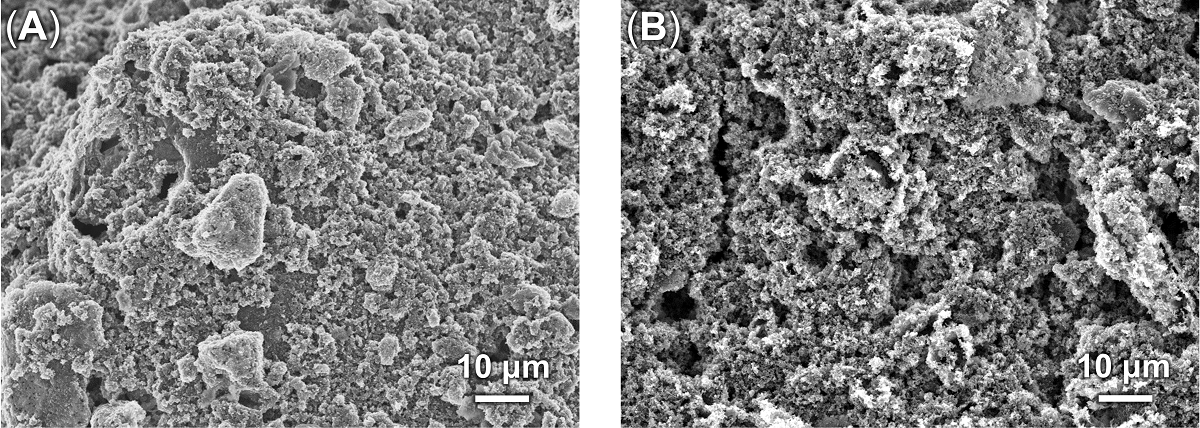


**Figure S15.** SEM images of fresh (A) S_8_ cathode, and (B) poly(S-*r*-Ccys) cathode.


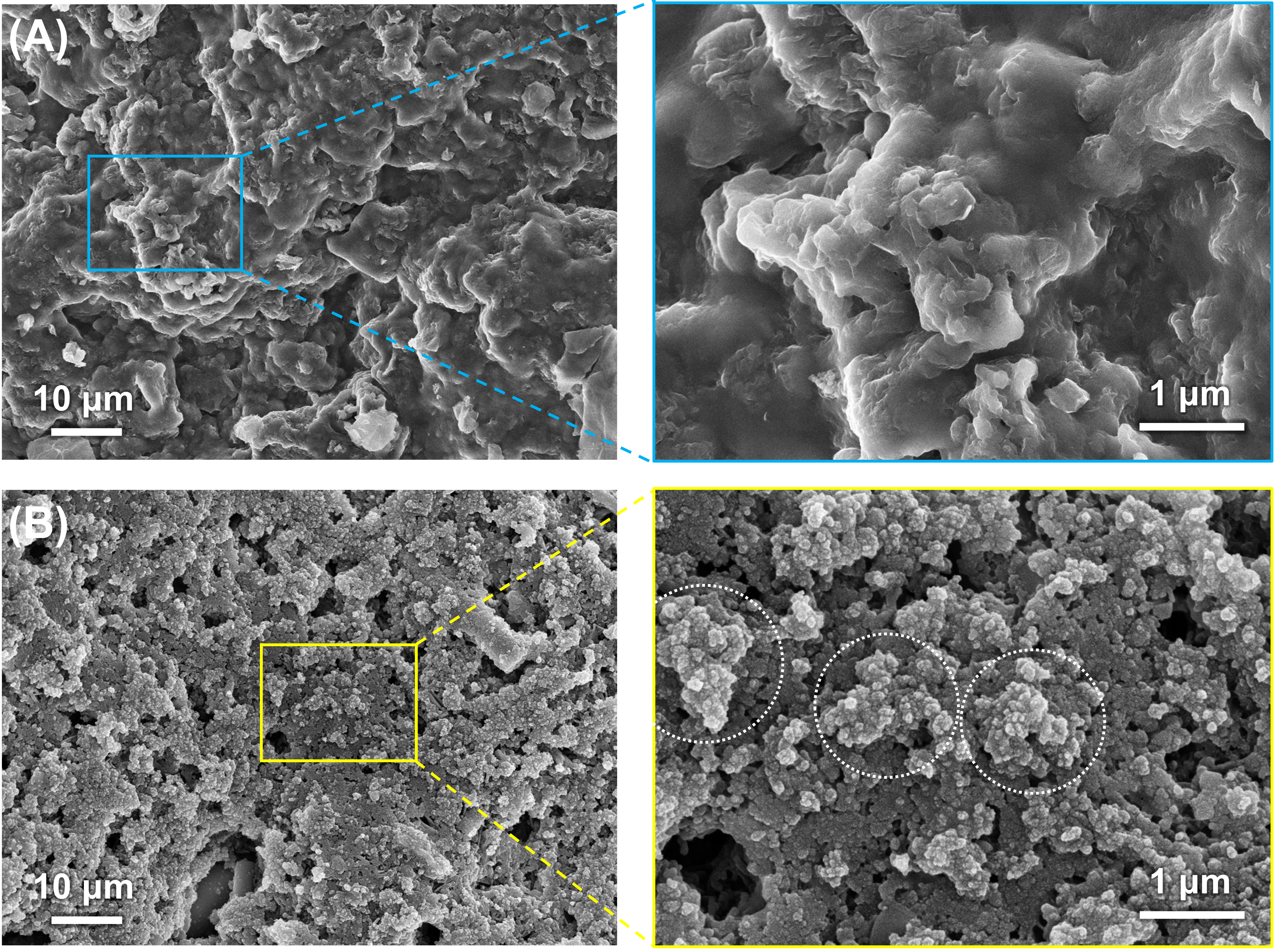


**Figure S16.** (A) SEM image of the S_8_ cathode at fully discharged state, (B) SEM image of the poly(S-*r*-Ccys) cathode at fully discharged state.


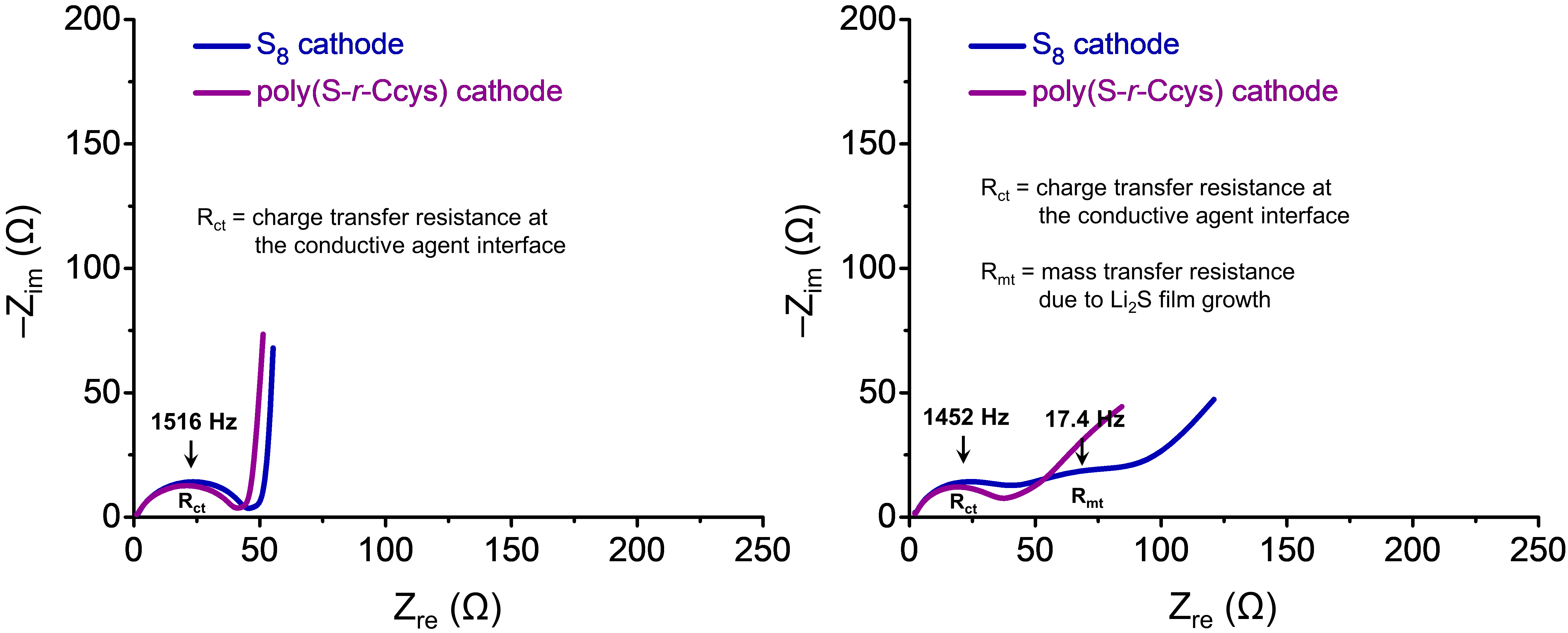


**Figure S17.** EIS spectra of the S_8_ and poly(S-r-Ccys) cathodes before (left) and after 100 cycles at a fully discharged state (right).


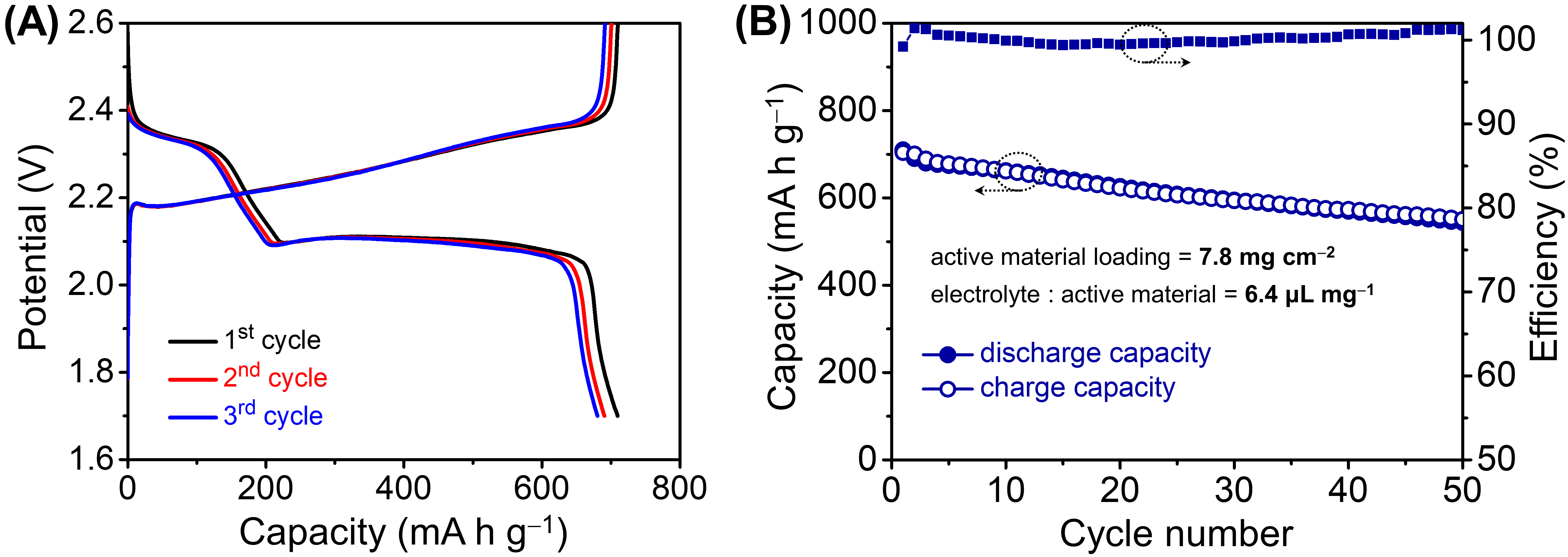


**Figure S18.** Galvanostatic cycling performance of the high-loading poly(S-*r*-Ccys) cathode under electrolyte-lean condition at a 0.1C rate.

**Computational Methods and Details:**

Following the reaction sequence, the relative free energy of the discharge process^[4]^ from S_8_ to Li_2_S on the substrate was estimated,

S_8_ + Li → Li_2_S_8­_ → Li_2_S_6­_ → Li_2_S_4_ → Li_2_S_2_ → Li_2_S

The corresponding reaction energies in all five steps were calculated^4^ from the equations:

$\Delta E_{1}=E_{Li_{2}S_{8}}-\left\{ E_{s_{8}}+2E_{Li} \right\}$ -------------------------------------------------------------(1)

$\Delta E_{2}=E_{Li_{2}S_{6}}+\frac{1}{4}E_{S_{8}}-E_{Li_{2}S_{8}}$ -------------------------------------------------------------(2)

$\Delta E_{3}=E_{Li_{2}S_{4}}+\frac{1}{4}E_{S_{8}}-E_{Li_{2}S_{6}}$ -------------------------------------------------------------(3)

$\Delta E_{4}=E_{Li_{2}S_{2}}+\frac{1}{4}E_{S_{8}}-E_{Li_{2}S_{4}}$ -------------------------------------------------------------(4)

$\Delta E_{5}=E_{Li_{2}S}+\frac{1}{8}E_{S_{8}}-E_{Li_{2}S_{2}}$ -------------------------------------------------------------(5)

Similarly, considering Li_2_S formation in each step the reaction for Lithium organo-polysulfides can be written as below:

Li(org-SLi)S_10_ → Li(org-SLi)S_9_ → ……………………… Li(org-SLi)S_2_ → Li(org-SLi)S

The corresponding reaction energies in all ten steps were calculated from the equations:

$\Delta E_{1}=E_{Li\left( org-SLi \right)S_{10}}-\left\{ E_{Li\left( org-S \right)}+{\frac{5}{4}E}_{s_{8}}+2E_{Li} \right\}$ -----------------------------------------------(6)

$\Delta E_{2}=E_{Li\left( org-SLi \right)S_{9}}+\frac{1}{8}E_{S_{8}}-E_{Li\left( org-SLi \right)S_{10}}$ -----------------------------------------------(7)

$\Delta E_{3}=E_{Li\left( org-SLi \right)S_{8}}+\frac{1}{8}E_{S_{8}}-E_{Li\left( org-SLi \right)S_{9}}$ -----------------------------------------------(8)

………………………………………………….

$\Delta E_{9}=E_{Li\left( org-SLi \right)S_{3}}+\frac{1}{8}E_{S_{8}}-E_{Li\left( org-SLi \right)S_{2}}$ ----------------------------------------------(14)

$\Delta E_{10}=E_{Li\left( org-SLi \right)S_{2}}+\frac{1}{8}E_{S_{8}}-E_{Li\left( org-SLi \right)S}$ ----------------------------------------------(15)

Where E_Li_ is the energy of lithium metal per atom and $E_{S_{8}}$ is the energy of S_8_.

To investigate the interactions between Li_2_S and the organo-polysulfide, Density Functional Theory (DFT) calculations were conducted on a theoretical model. Separate optimizations of Li_2_S near the 'O' and 'N' atoms of the copolymer were performed.

**Computational Investigations**

To investigate the potential reason for the greater activity of lithium organo-polysulfides than that of lithium polysulfides, calculations based on the density functional theory (DFT) were carried out. For simplicity, we used the truncated monomeric model of lithium organo-polysulfides as shown in **Figure S19**, in which only one side chain of polysulfur is considered.


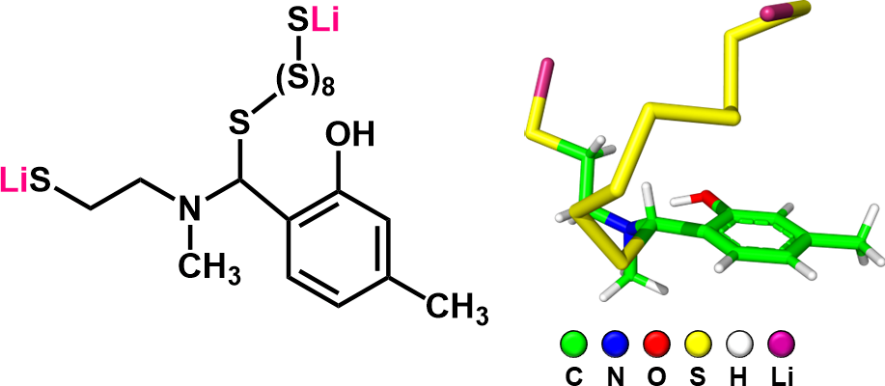


**Figure S19.** 2D and 3D structure of the truncated monomeric model of lithium organo-polysulfides. The color code for atoms is shown in the bottom right.

First, the highest occupied molecular orbital (HOMO) and lowest unoccupied molecular orbital (LUMO) energy levels and the band gaps for different S species were computed similarly to the previous calculations.^[5]^


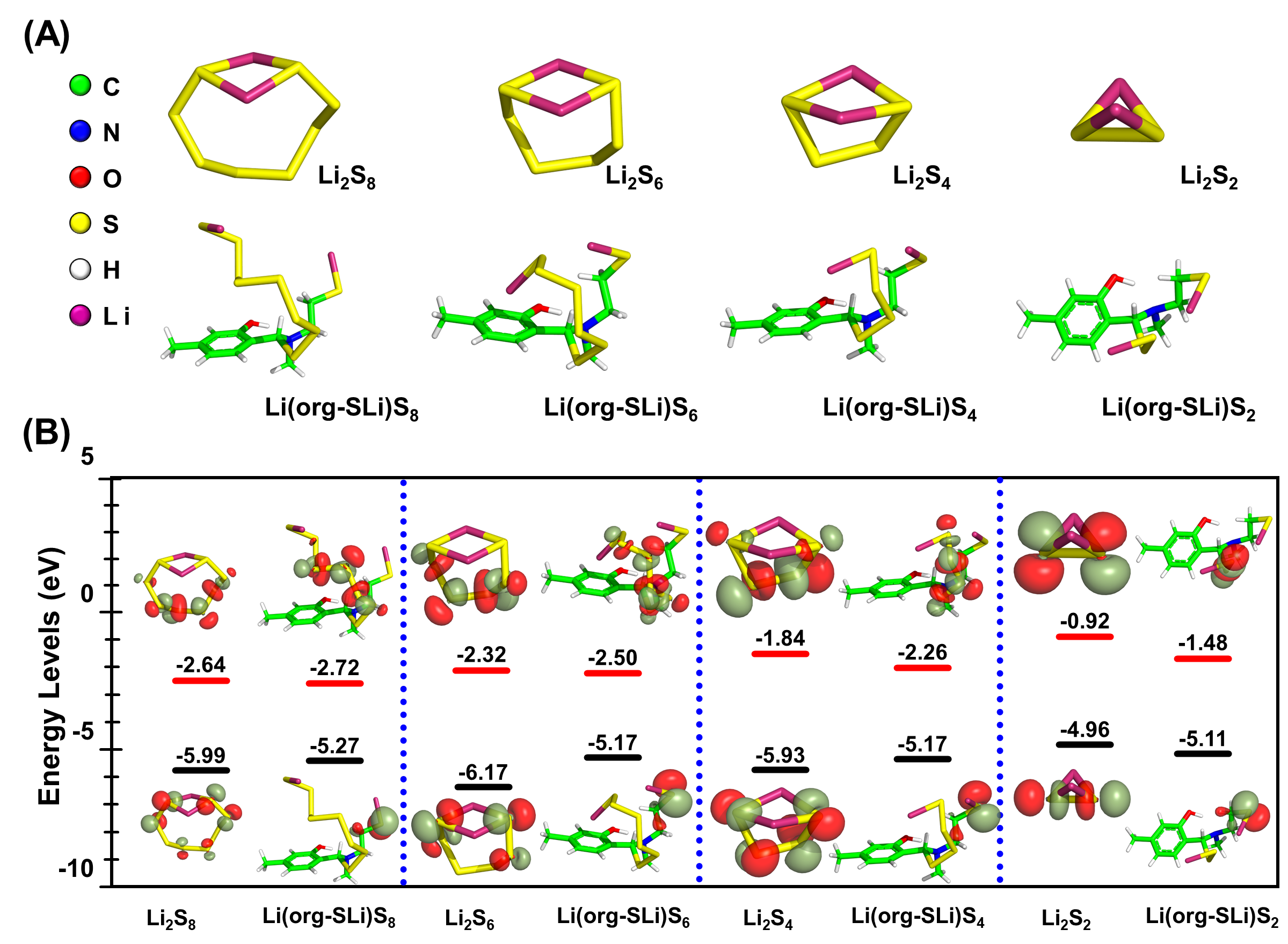


**Figure S20.** (A) The optimized structures of different lithium polysulfides and lithium organo-polysulfides. The atom color codes are on the left, (B) The comparison of the lowest unoccupied molecular orbital (LUMO) and the highest occupied molecular orbital (HOMO) energy levels of different states. Note that red and black are LUMO and HOMO energy levels, respectively. The isosurfaces for energy levels are drawn with an isovalue of 0.05.

Additionally, we estimated the relative Gibbs free energy^[4]^ (G) throughout the reduction process to further our knowledge of increased redox kinetics considering the formation of Li_2_S in each step. **Figure S20** shows the relative free energy profile for the discharging process from S_8_ to end-discharged S products in Li-polysulfides and lithium organo-polysulfides. The optimized intermediate states are shown in the inset and below the profile. The relative Gibbs free energies have been quantified from the reaction energies of each step.

Specifically, the conversion of 1 from S_8_ is relatively more exothermic. Li_2_S_2_→Li_2_S precipitation appears to be the rate-limiting step during discharging since the final two processes from Li_2_S_4_ to Li_2_S_2_ and from Li_2_S_2_ to Li_2_S showed the highest energy barriers (0.79 and 0.85 eV each) than the preceding steps; however, barrier for the last step in lithium organo-polysulfides is lowered to 0.72 eV for the ultimate reduction of S_8_→10. This conclusion suggests that the final discharging step is more favorable for lithium organo-polysulfides from a thermodynamic standpoint.


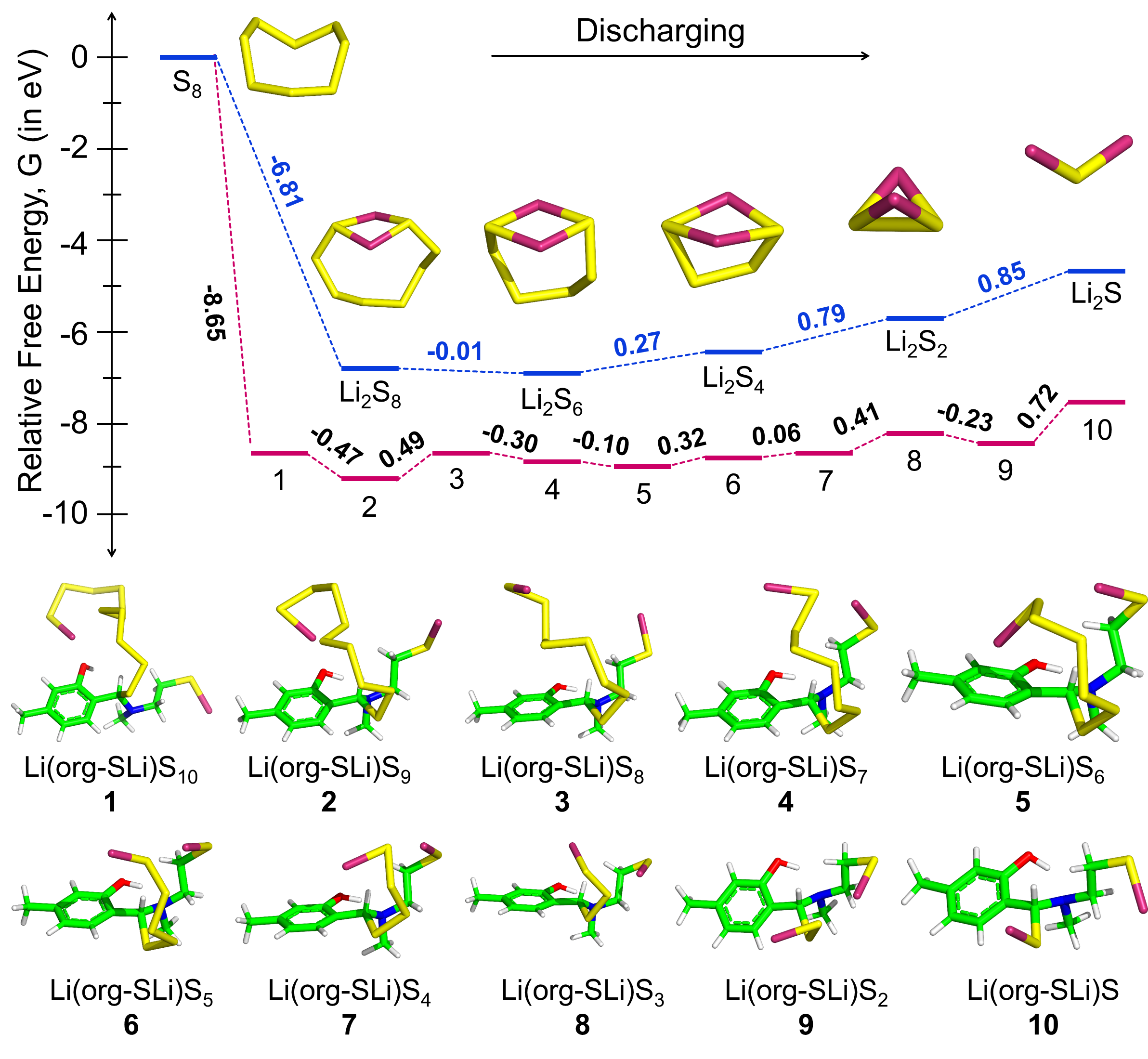


**Figure S21.** Relative free energy for the discharging process of S_8_ for both lithium polysulfides and lithium organo-polysulfides. The optimized structures of the Li_2_S_n_ intermediates are as the insets and lithium organo-polysulfides are given at the bottom.

**Cartesian coordinates for QM optimized geometry:**

***“Lithium Organo Polysulfides”*:**

**1**

C -2.218000 4.487000 -0.297000

C -3.462000 3.853000 -0.176000

C -3.566000 2.469000 -0.222000

C -2.44 1.65 -0.387000

C -1.21 2.296000 -0.53

C -1.094000 3.683000 -0.484000

H -4.359000 4.449000 -0.042000

H -4.546000 2.015000 -0.119000

H -0.112000 4.127000 -0.613000

O -0.025000 1.564000 -0.704000

H -0.043000 1.077000 -1.54

C -2.098000 5.987000 -0.218000

H -1.161000 6.337000 -0.655000

C -2.557000 0.141000 -0.375000

H -1.563000 -0.313000 -0.314000

S -3.355000 -0.314000 1.306000

N -3.386000 -0.388000 -1.446000

C -3.145000 0.237000 -2.755000

H -3.798000 -0.244000 -3.488000

H -3.40 1.296000 -2.734000

C -3.424000 -1.864000 -1.53

H -3.708000 -2.253000 -0.551000

S -2.139000 -1.714000 2.212000

S -0.759000 -0.586000 3.473000

S 1.156000 -1.339000 3.199000

S 2.179000 -0.323000 1.641000

S 2.038000 -1.548000 -0.124000

S 4.023000 -2.065000 -0.533000

S 4.826000 -0.512000 -1.774000

S 5.229000 1.18 -0.578000

S 3.661000 2.574000 -0.742000

C -2.149000 -2.573000 -2.006000

H -1.304000 -2.309000 -1.367000

S -2.299000 -4.422000 -1.957000

Li 1.657000 1.752000 0.159000

H -2.925000 6.479000 -0.736000

H -2.123000 6.323000 0.825000

H -4.247000 -2.112000 -2.209000

H -1.898000 -2.267000 -3.023000

H -2.11 0.14 -3.114000

Li -4.182000 -4.955000 -3.047000

**2**

C 0.51 4.208000 -0.814000

C -0.725000 4.129000 -0.168000

C -1.471000 2.95 -0.181000

C -1.018000 1.808000 -0.84

C 0.222000 1.904000 -1.496000

C 0.972000 3.071000 -1.487000

H -1.115000 4.994000 0.357000

H -2.418000 2.924000 0.341000

H 1.92 3.088000 -2.014000

O 0.744000 0.797000 -2.156000

H -0.035000 0.246000 -2.437000

C 1.335000 5.469000 -0.794000

H 1.601000 5.785000 -1.808000

C -1.735000 0.461000 -0.876000

H -1.156000 -0.267000 -0.31

S -3.384000 0.495000 0.105000

N -1.857000 -0.034000 -2.235000

C -2.664000 0.823000 -3.123000

H -3.74 0.72 -2.929000

H -2.386000 1.87 -2.999000

C -2.259000 -1.458000 -2.367000

H -3.224000 -1.626000 -1.873000

S -3.045000 -0.746000 1.743000

S -2.085000 0.433000 3.262000

S -0.189000 -0.386000 3.673000

S 1.21 0.285000 2.24

S 1.355000 -1.339000 0.859000

S 3.27 -2.214000 1.052000

S 4.227000 -1.564000 -0.769000

S 4.54 0.496000 -0.709000

C -1.226000 -2.458000 -1.855000

H -1.088000 -2.358000 -0.778000

S -1.745000 -4.198000 -2.243000

H 0.797000 6.288000 -0.313000

H 2.272000 5.315000 -0.248000

H -2.414000 -1.632000 -3.433000

H -0.262000 -2.267000 -2.334000

H -2.477000 0.541000 -4.16

Li -1.527000 -5.233000 -0.267000

Li 2.426000 0.096000 -1.589000

**3**

C -1.183000 -4.051000 0.537000

C -0.882000 -3.284000 1.666000

C 0.073000 -2.272000 1.604000

C 0.774000 -1.982000 0.43

C 0.466000 -2.765000 -0.709000

C -0.492000 -3.776000 -0.644000

H -1.398000 -3.47 2.602000

H 0.27 -1.689000 2.494000

H -0.688000 -4.344000 -1.549000

O 1.072000 -2.561000 -1.919000

H 1.848000 -1.966000 -1.747000

C -2.218000 -5.146000 0.581000

H -1.791000 -6.109000 0.281000

C 1.742000 -0.808000 0.291000

H 1.211000 0.048000 -0.128000

S 2.302000 -0.149000 2.014000

N 2.863000 -1.108000 -0.574000

C 3.788000 -2.132000 -0.063000

H 4.469000 -1.733000 0.699000

H 3.231000 -2.965000 0.368000

C 3.577000 0.069000 -1.122000

H 3.888000 0.745000 -0.314000

S 1.96 1.904000 1.939000

S 0.144000 2.349000 2.963000

S -1.368000 2.829000 1.589000

S -2.557000 1.086000 1.232000

S -1.949000 0.173000 -0.586000

S -3.00 1.256000 -2.097000

S -5.029000 0.707000 -2.02

C 2.779000 0.829000 -2.18

H 1.861000 1.238000 -1.755000

S 3.79 2.219000 -2.883000

H -2.634000 -5.259000 1.585000

H -3.046000 -4.935000 -0.104000

H 4.492000 -0.309000 -1.58

H 2.498000 0.135000 -2.977000

H 4.389000 -2.515000 -0.891000

Li 2.496000 2.999000 -4.54

Li -4.641000 2.098000 -0.253000

**4**

C -4.345000 -1.607000 0.793000

C -3.679000 -1.924000 -0.394000

C -2.673000 -1.095000 -0.886000

C -2.289000 0.081000 -0.234000

C -2.961000 0.389000 0.974000

C -3.969000 -0.442000 1.464000

H -3.942000 -2.822000 -0.942000

H -2.173000 -1.378000 -1.802000

H -4.455000 -0.154000 2.391000

O -2.65 1.49 1.725000

H -2.047000 2.047000 1.162000

C -5.441000 -2.485000 1.342000

H -6.385000 -1.936000 1.425000

C -1.114000 0.945000 -0.699000

H -0.201000 0.59 -0.22

S -0.765000 0.647000 -2.571000

N -1.277000 2.349000 -0.375000

C -2.304000 3.06 -1.153000

H -1.961000 3.307000 -2.165000

H -3.207000 2.451000 -1.23

C -0.002000 3.101000 -0.265000

H 0.662000 2.868000 -1.106000

S 1.295000 0.437000 -2.775000

S 1.783000 -1.647000 -2.757000

S 2.98 -2.054000 -1.072000

S 1.841000 -2.803000 0.541000

S 1.286000 -1.158000 1.777000

S 2.949000 -0.537000 2.942000

C 0.702000 2.838000 1.067000

H 0.754000 1.76 1.241000

S 2.408000 3.564000 1.132000

H -5.612000 -3.353000 0.702000

H -5.192000 -2.849000 2.345000

H -0.241000 4.164000 -0.341000

H 0.099000 3.258000 1.877000

H -2.563000 3.988000 -0.639000

Li 3.292000 1.666000 2.003000

Li 1.583000 -1.878000 4.206000

**5**

C -3.91 -1.573000 -0.018000

C -2.879000 -1.961000 -0.89

C -1.843000 -1.078000 -1.197000

C -1.77 0.217000 -0.66

C -2.826000 0.615000 0.203000

C -3.855000 -0.286000 0.525000

H -2.88 -2.953000 -1.327000

H -1.051000 -1.42 -1.852000

H -4.621000 0.056000 1.212000

O -2.872000 1.831000 0.787000

H -2.055000 2.324000 0.456000

C -5.05 -2.501000 0.303000

H -5.848000 -2.389000 -0.439000

C -0.511000 1.075000 -0.814000

H 0.223000 0.691000 -0.104000

S 0.323000 0.743000 -2.498000

N -0.718000 2.489000 -0.518000

C -1.245000 3.30 -1.626000

H -0.484000 3.515000 -2.384000

H -2.08 2.783000 -2.107000

C 0.445000 3.095000 0.184000

H 1.383000 2.758000 -0.27

S 2.339000 0.387000 -2.071000

S 2.654000 -1.693000 -1.763000

S 3.05 -2.049000 0.304000

S 1.412000 -2.851000 1.341000

S 0.323000 -1.303000 2.299000

C 0.412000 2.756000 1.684000

H -0.059000 1.777000 1.814000

S 2.076000 2.742000 2.505000

H -4.73 -3.546000 0.283000

H -5.479000 -2.286000 1.284000

H 0.397000 4.176000 0.043000

H -0.237000 3.468000 2.199000

H -1.618000 4.246000 -1.228000

Li 1.895000 0.488000 2.78

Li -1.728000 -1.431000 1.12

**6**

C -4.511000 -1.233000 0.909000

C -3.555000 -2.058000 0.305000

C -2.388000 -1.517000 -0.222000

C -2.107000 -0.143000 -0.179000

C -3.063000 0.678000 0.464000

C -4.241000 0.131000 0.983000

H -3.723000 -3.128000 0.242000

H -1.67 -2.186000 -0.677000

H -4.941000 0.809000 1.46

O -2.888000 2.022000 0.64

H -2.034000 2.25 0.178000

C -5.804000 -1.796000 1.443000

H -6.576000 -1.804000 0.664000

C -0.767000 0.418000 -0.665000

H -0.003000 0.226000 0.089000

S -0.187000 -0.659000 -2.163000

N -0.792000 1.843000 -0.936000

C -1.449000 2.244000 -2.189000

H -0.818000 2.06 -3.067000

H -2.386000 1.698000 -2.315000

C 0.505000 2.53 -0.701000

H 1.335000 1.938000 -1.099000

S 1.892000 -0.746000 -2.144000

S 2.481000 -2.415000 -0.905000

S 3.693000 -1.672000 0.636000

S 2.521000 -0.905000 2.237000

C 0.722000 2.835000 0.783000

H 0.50 1.946000 1.381000

S 2.442000 3.427000 1.152000

H -5.679000 -2.826000 1.787000

H -6.187000 -1.20 2.275000

H 0.49 3.466000 -1.262000

H 0.01 3.603000 1.098000

H -1.681000 3.31 -2.144000

Li 3.094000 1.386000 1.888000

Li 2.399000 -2.81 3.526000

**7**

C -4.555000 -0.693000 0.658000

C -3.765000 -1.607000 -0.048000

C -2.477000 -1.267000 -0.452000

C -1.913000 -0.013000 -0.183000

C -2.718000 0.904000 0.535000

C -4.009000 0.558000 0.941000

H -4.155000 -2.591000 -0.288000

H -1.898000 -2.006000 -0.99

H -4.578000 1.301000 1.49

O -2.279000 2.15 0.884000

H -1.431000 2.297000 0.384000

C -5.963000 -1.034000 1.073000

H -6.675000 -0.769000 0.282000

C -0.462000 0.35 -0.515000

H 0.163000 0.128000 0.349000

S 0.208000 -0.876000 -1.852000

N -0.293000 1.752000 -0.835000

C -0.889000 2.178000 -2.11

H -0.27 1.894000 -2.97

H -1.88 1.737000 -2.235000

C 1.085000 2.275000 -0.653000

H 1.804000 1.666000 -1.21

S 2.262000 -1.07 -1.496000

S 2.67 -2.622000 -0.087000

S 2.833000 -1.77 1.867000

C 1.497000 2.384000 0.818000

H 1.197000 1.489000 1.369000

S 3.322000 2.645000 1.035000

H -6.075000 -2.104000 1.265000

H -6.26 -0.49 1.973000

H 1.109000 3.27 -1.104000

H 0.964000 3.219000 1.28

H -1.001000 3.265000 -2.107000

Li 3.73 0.454000 1.477000

Li 0.785000 -2.756000 1.668000

**8**

C -4.289000 0.201000 -0.455000

C -3.583000 1.216000 0.197000

C -2.235000 1.054000 0.515000

C -1.536000 -0.117000 0.209000

C -2.258000 -1.142000 -0.448000

C -3.605000 -0.976000 -0.768000

H -4.083000 2.143000 0.458000

H -1.714000 1.863000 1.009000

H -4.111000 -1.794000 -1.272000

O -1.672000 -2.325000 -0.815000

H -0.802000 -2.367000 -0.331000

C -5.749000 0.352000 -0.799000

H -6.368000 -0.32 -0.193000

C -0.032000 -0.285000 0.433000

H 0.482000 -0.064000 -0.504000

S 0.684000 1.04 1.611000

N 0.321000 -1.649000 0.809000

C -0.151000 -2.055000 2.142000

H 0.448000 -1.613000 2.948000

H -1.193000 -1.759000 2.282000

C 1.75 -1.967000 0.601000

H 2.374000 -1.21 1.095000

S 2.288000 1.922000 0.551000

S 1.617000 3.359000 -0.847000

C 2.129000 -2.111000 -0.88

H 1.543000 -1.425000 -1.495000

S 3.931000 -1.815000 -1.208000

H -6.096000 1.372000 -0.622000

H -5.94 0.104000 -1.848000

H 1.956000 -2.907000 1.118000

H 1.875000 -3.118000 -1.221000

H -0.092000 -3.142000 2.226000

Li 3.905000 0.32 -0.444000

Li 0.728000 2.10 -2.55

**9**

C -4.206000 -0.112000 -0.398000

C -3.644000 1.049000 0.137000

C -2.27 1.133000 0.37

C -1.408000 0.074000 0.085000

C -1.99 -1.113000 -0.416000

C -3.359000 -1.192000 -0.663000

H -4.278000 1.896000 0.378000

H -1.871000 2.047000 0.793000

H -3.752000 -2.123000 -1.06

O -1.232000 -2.223000 -0.685000

H -0.383000 -2.119000 -0.187000

C -5.685000 -0.216000 -0.675000

H -6.131000 -1.062000 -0.142000

C 0.112000 0.12 0.206000

H 0.536000 0.106000 -0.799000

S 0.773000 1.807000 0.862000

N 0.629000 -1.035000 0.919000

C 0.156000 -1.165000 2.305000

H 0.634000 -0.441000 2.978000

H -0.925000 -1.023000 2.354000

C 2.099000 -1.192000 0.83

H 2.585000 -0.264000 1.162000

S 2.133000 2.475000 -0.661000

C 2.608000 -1.605000 -0.555000

H 2.066000 -1.081000 -1.345000

S 4.426000 -1.277000 -0.756000

H -6.211000 0.691000 -0.369000

H -5.877000 -0.372000 -1.742000

H 2.386000 -1.957000 1.555000

H 2.424000 -2.671000 -0.707000

H 0.384000 -2.17 2.664000

Li 3.995000 0.961000 -0.739000

Li 0.031000 3.307000 -0.913000

**10**

C -4.015000 -0.418000 -0.26

C -3.614000 0.92 -0.222000

C -2.261000 1.255000 -0.132000

C -1.259000 0.287000 -0.078000

C -1.676000 -1.064000 -0.095000

C -3.024000 -1.403000 -0.189000

H -4.358000 1.708000 -0.259000

H -1.964000 2.297000 -0.101000

H -3.287000 -2.457000 -0.197000

O -0.764000 -2.086000 -0.016000

H 0.08 -1.662000 0.326000

C -5.47 -0.802000 -0.368000

H -5.764000 -1.482000 0.439000

C 0.241000 0.608000 -0.096000

H 0.599000 0.297000 -1.079000

S 0.681000 2.424000 0.069000

N 0.952000 -0.276000 0.861000

C 0.618000 -0.017000 2.268000

H 1.04 0.93 2.63

H -0.465000 0.019000 2.401000

C 2.417000 -0.33 0.683000

H 2.844000 0.67 0.855000

C 2.886000 -0.867000 -0.669000

H 2.561000 -0.217000 -1.483000

S 4.735000 -1.018000 -0.759000

H -6.117000 0.076000 -0.319000

H -5.677000 -1.318000 -1.312000

H 2.799000 -0.988000 1.468000

H 2.454000 -1.856000 -0.843000

H 1.009000 -0.832000 2.882000

Li 5.387000 0.857000 0.291000

Li 0.604000 3.122000 -2.062000

**Lithium Polysulfides:**

**Li_2_S_8_**

Li -0.207000 1.925000 1.467000

Li 0.204000 1.932000 -1.461000

S -1.827000 2.306000 -0.254000

S -3.011000 0.55 -0.379000

S -2.239000 -0.957000 0.841000

S -0.987000 -2.262000 -0.344000

S 0.989000 -2.258000 0.351000

S 2.236000 -0.956000 -0.845000

S 3.015000 0.552000 0.369000

S 1.825000 2.303000 0.261000

**Li_2_S_6_**

Li -1.368000 -0.301000 1.588000

Li 1.368000 0.301000 1.588000

S -0.354000 1.908000 1.636000

S 0.684000 2.068000 -0.214000

S -0.354000 1.005000 -1.72

S 0.354000 -1.005000 -1.72

S -0.684000 -2.068000 -0.214000

S 0.354000 -1.908000 1.636000

**Li_2_S_4_**

Li 1.422000 0.327000 -1.139000

Li -1.422000 -0.327000 -1.139000

S -0.374000 1.836000 -0.804000

S 0.374000 1.012000 1.017000

S -0.374000 -1.012000 1.017000

S 0.374000 -1.836000 -0.804000

**Li_2_S_2_**

Li -0.00 1.688000 0.892000

Li 0.00 -1.688000 0.892000

S 1.115000 0.00 -0.167000

S -1.115000 -0.00 -0.167000

**Li_2_S**

Li 0.00 1.881000 -0.655000

Li 0.00 -1.881000 -0.655000

S 0.00 0.0000 0.249000

**S_8_**

S -2.212000 0.072000 0.923000

S -1.765000 1.466000 -0.569000

S -1.759000 -1.897000 0.106000

S -0.00 2.445000 0.083000

S 0.00 -1.728000 -1.003000

S 1.764000 1.466000 -0.569000

S 1.759000 -1.897000 0.106000

S 2.212000 0.073000 0.923000

**Li_2_S in the Vicinity of ‘O’ of Organo Polysulfide**

C 2.3390 4.2650 -0.9240

C 3.5430 3.6420 -0.5570

C 3.5640 2.3090 -0.1770

C 2.3910 1.5270 -0.1510

C 1.1970 2.1450 -0.5760

C 1.1710 3.4900 -0.9310

H 4.4680 4.2170 -0.5380

H 4.5030 1.8530 0.1390

H 0.2090 3.9470 -1.1580

O -0.0790 1.4980 -0.5540

H -0.0080 0.6100 -0.9290

C 2.2980 5.7330 -1.2700

H 1.3340 6.1720 -1.000

C 2.4940 0.0810 0.2870

H 1.5130 -0.4170 0.2880

S 3.4490 -0.7860 -1.1280

N 3.2520 -0.0820 1.5090

C 2.8130 0.7710 2.6260

H 3.5680 0.6980 3.4230

H 2.7410 1.8240 2.3190

C 3.5390 -1.4700 1.9210

H 4.0490 -1.9740 1.1000

S 2.3250 -2.3910 -1.7750

S 0.9600 -1.5990 -3.2850

S -0.9370 -2.3650 -2.9310

S -2.0610 -1.1050 -1.6460

S -1.9520 -1.9460 0.3330

S -3.9330 -2.4580 0.7680

S -4.8440 -0.7250 1.6430

S -5.2560 0.6750 0.1170

S -3.7480 2.1420 0.0530

C 2.3420 -2.3300 2.3560

H 1.5620 -2.2980 1.5900

S 2.7720 -4.1220 2.5480

Li -2.5150 2.0450 -1.9410

H 3.0780 6.2790 -0.7330

H 2.4590 5.9030 -2.3420

H 4.2630 -1.4050 2.7430

H 1.9040 -1.9540 3.2820

H 1.8310 0.4760 3.0410

Li 4.2560 -4.4250 4.0950

S -0.6330 3.9570 2.3190

Li -1.1890 2.0420 1.1610

Li -1.8990 5.4210 1.6000

**Li_2_S in the Vicinity of ‘N’ of Organo Polysulfide**

C -2.3230 4.3340 0.1640

C -3.4070 3.4510 -0.0860

C -3.1950 2.0890 -0.0870

C -1.9230 1.5120 0.1390

C -0.8750 2.3750 0.4120

C -1.0890 3.7570 0.4090

H -4.3880 3.8370 -0.2830

H -4.0670 1.4820 -0.2890

H -0.2300 4.3840 0.6060

O 0.4300 2.0370 0.7000

H 0.5990 1.0830 0.6560

C -2.5290 5.8330 0.1830

H -1.6160 6.3660 -0.0420

C -1.8360 -0.0330 0.1250

H -0.8100 -0.3740 0.3040

S -2.7520 -0.7000 1.6510

N -2.3950 -0.5970 -1.1330

C -1.6050 -0.1170 -2.3020

H -2.0230 -0.5380 -3.1940

H -1.6730 0.9560 -2.4010

C -2.5920 -2.0890 -1.1770

H -3.1300 -2.3510 -0.2750

S -1.3090 -1.9170 2.4840

S 0.0690 -0.5860 3.5310

S 2.0140 -1.1610 3.0850

S 2.7570 -0.1410 1.3780

S 2.5620 -1.4750 -0.3010

S 4.5370 -1.8160 -0.8990

S 5.0400 -0.2660 -2.2930

S 5.3850 1.5240 -1.2300

S 3.6720 2.7450 -1.2880

C -1.3810 -3.0170 -1.3490

H -0.6470 -2.8540 -0.5450

S -1.8750 -4.7900 -1.2470

Li 2.7100 3.0080 0.8360

H -3.2890 6.1300 -0.5370

H -2.8870 6.1450 1.1790

H -3.2590 -2.2680 -2.0450

H -0.8770 -2.8410 -2.2940

H -0.5490 -0.3900 -2.2450

Li -3.5370 -5.6460 -2.3600

S -6.4090 0.2860 -1.6410

Li -4.3610 -0.3350 -1.5360

Li -8.2700 0.7410 -2.458

**Supplementary Note 1.**

Δ*E*_interaction_ = *E*_complex_ − (*E*_polymer_ + *E*_Li2S_)

Li–O complex: Δ*E*_interaction_ = −0.034741 Hartree ≈ −21.80 kcal mol^−1^

Li–N complex: Δ*E*_interaction_ = −0.042546 Hartree ≈ −26.69 kcal mol^−1^

**Supplementary Note 2.**

Randles–Sevcik equation for lithium-ion diffusion coefficient test:

𝐼_p_ = 2.69 × 10^5^𝑛^1.5^𝐴(𝐷_Li_^+^)^0.5^𝐶_Li_^+^𝑣^0.5^

𝐼_p_ is the peak current in *ampere*, *n* indicates the number of electrons in the redox reaction (e.g., n = 2 for sulfur redox reactions), A represents the electrode area in *cm^2^*, 𝐷_Li_^+^ is the lithium-ion diffusion coefficient in *cm^2^ s^–1^*, C_Li_^+^ indicates the lithium-ion concentration in the electrolyte in *molarity*, and *v* is the scanning rate in *V s^–1^*.

**References**

[1] W. Bao, D. Su, W. Zhang, X. Guo, G. Wang, *Adv. Funct. Mater.* **2016**, *26*, 8746.

[2] X. Tao, J. Wang, C. Liu, H. Wang, H. Yao, G. Zheng, Z. W. Seh, Q. Cai, W. Li, G. Zhou, C. Zu, Y. Cui, *Nat. Commun.* **2016**, *7*, 11203.

[3] M. Zhao, X. Chen, X.-Y. Li, B.-Q. Li, J.-Q. Huang, *Adv. Mater.* **2021**, *33*, 2007298.

[4] C. Zhao, G.-L. Xu, Z. Yu, L. Zhang, I. Hwang, Y.-X. Mo, Y. Ren, L. Cheng, C.-J. Sun, Y. Ren, X. Zuo, J.-T. Li, S.-G. Sun, K. Amine, T. Zhao, *Nat. Nanotechnol.* **2021**, *16*, 166.

[5] C. Jiang, L. Li, Q. Jia, M. Tang, K. Fan, Y. Chen, C. Zhang, M. Mao, J. Ma, W. Hu, C. Wang, *ACS Nano* **2022**, *16*, 9163.
